# Rapid, Highly Sustainable Ring-Opening Polymerization via Resonant Acoustic Mixing

**DOI:** 10.1021/acssuschemeng.4c06330

**Published:** 2025-01-31

**Authors:** Harriet R. Fowler, Riley O’Shea, Joseph Sefton, Shaun C. Howard, Benjamin W. Muir, Robert A. Stockman, Vincenzo Taresco, Derek J. Irvine

**Affiliations:** † School of Chemistry, University of Nottingham, University Park, Nottingham NG7 2RD, U.K.; ‡ CSIRO Manufacturing, Clayton, Victoria 3168, Australia; § Centre for Additive Manufacturing, Department of Chemical and Environmental Engineering, Faculty of Engineering, University of Nottingham, University Park, Nottingham NG7 2RD, U.K.

**Keywords:** resonant acoustic mixing, high throughput, terpenes, biodegradable polymers, polyesters, drug encapsulation

## Abstract

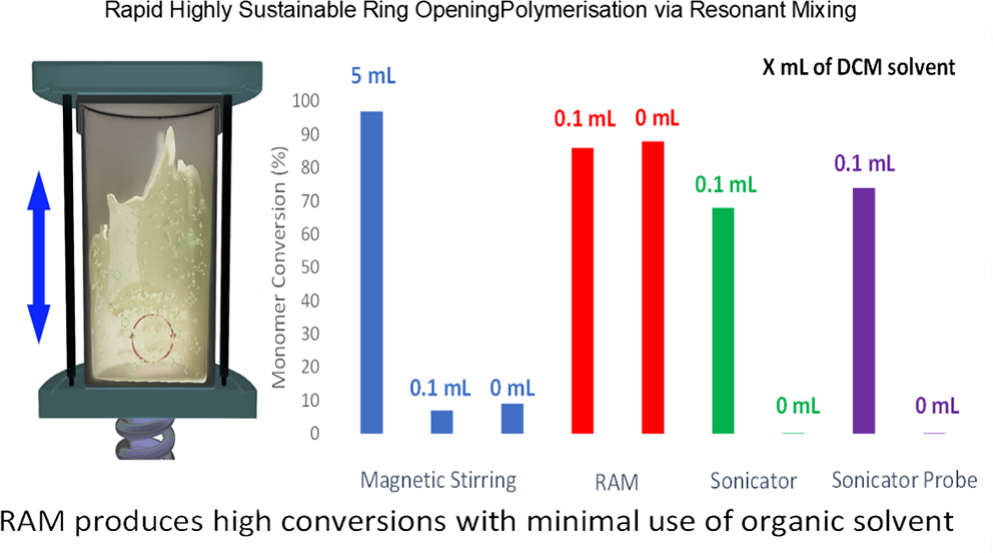

Reported herein is the first combination of resonant acoustic mixing (RAM) and controlled ring-opening polymerization (ROP) to deliver fully sustainable, end-functionalized, biodegradable polymers via a manufacturing route with a much-reduced environmental impact. This includes the successful use of agriculturally sourced functionalized initiators (terpene alcohols) in ROP synthesis of cyclic esters to generate an array of novel, biodegradable polyesters applicable to numerous biomedical applications, such as drug delivery. Furthermore, RAM was utilized as a novel mixing technique, resulting in a synthetic process that was conducted: (a) with minimal use of toxic, flammable, costly, and environmentally detrimental solvents, (b) in the absence of organometallic catalysts, and (c) with significantly shorter ROP reaction times and temperatures. Consequent comparison with conventional magnetic stirring or sonication-based mixing methods showed that RAM allowed the more facile, kilogram-scale synthesis of polyesters via reactions conducted at room temperature rather than 150 °C and without the need for a metal catalyst. As a proof of concept, the polymers were used to encapsulate bovine serum albumin as a model protein, and its release was measured using an automated, high-throughput protein assay. This study demonstrated that the headgroup chemistry appears to affect the release rate of protein from the polymers.

## Abstract 2

Resonance acoustic mixing methods synthesized biodegradable polymers with (a) no/minimal solvent, (b) no toxic catalysts, and (c) significantly reduced reaction times/temperatures.

## Introduction

Biodegradable polyesters have been extensively studied due to their potential to replace petrochemical-derived materials in a number of industries.^1,2^ The most widely studied biodegradable polyesters are poly(lactide) (PLA), polyglycolide (PGA), and poly(ε-caprolactone) (PCL), all of which have desirable properties and can be derived from renewable feedstocks.^1−3^ Among these, PLA-derived materials have received specific attention due to their biocompatibility, allowing them to find use in numerous applications ranging from controlled drug delivery to medical implants and surgical sutures.^4,5^ The lactic acid monomer can be obtained via bacterial fermentation of starch and other polysaccharides, which are easily sourced and inexpensive raw materials.^6−9^ Consequently, PLA is recognized as being a viable biodegradable/compostable polymer that breaks down to form lactic acid.^2,5^

One major drawback with PLA is the lack of chemical functionality on the polymer backbone, which can limit its broader utility.^10^ However, especially with low molecular weight polymers, this can be overcome by using functionalized initiators in ring-opening polymerization (ROP).^10,11^ These initiators become the “head” group of the polymer, thus introducing functionality. An example is the use of pegylated initiators to produce degradable, amphiphilic, block copolymer materials, which can be used for numerous biomedical applications.^10,12,13^ Alternatively, they may impart a range of functionalities that allow for postpolymerization modifications, e.g., hydroxyl end-groups for further reaction, (meth)acrylate centers for secondary polymerization, and stimuli-responsive groups in temperature-sensitive polymers.^10,14^

Terpenes and terpenoids are an attractive alternative feedstock to produce renewable polymers, due to their abundance from waste materials, low cost, and structural diversity.^15−17^ Thus, the use of monoterpene alcohols as initiators for the ROP of lactide has been explored by the authors.^5^ These materials were shown to self-assemble into well-defined nanoparticles (NPs), which were biocompatible and have potential for use as surfactants for drug delivery.^5^ Therefore, in this work, we introduce the first reported use of resonant acoustic mixing (RAM) for the ROP of cyclic esters in order to manufacture oligomeric surfactants of this type via a highly sustainable methodology.

**Figure 1:**
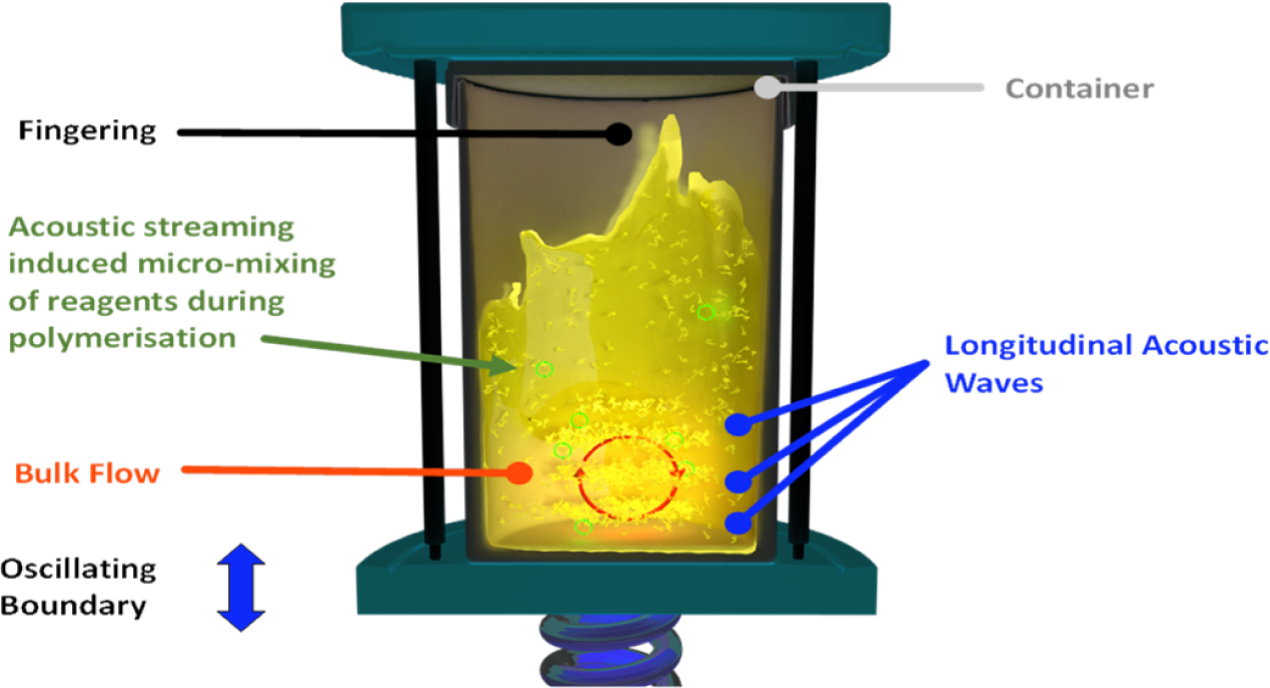
A schematic diagram showing the mixing processes occurring in the RAM.

RAM processing uses low-frequency, high-intensity acoustic agitation to facilitate the movement of the material and induce mixing. Thus, within the reaction vessel, in addition to conventional bulk flow mixing, the acoustic waves create micromixing zones. Consequently, forces of up to 100 G (i.e., acceleration due to gravity) can be imparted on the materials.^18−20^ The RAM operates at a resonant frequency of ∼60 Hz, such that the potential energy stored in the springs can be efficiently transferred to the plates. This energy is then transferred into the reaction vessel through the propagation of acoustic pressure waves.^18−20^ The mixing conditions are frequently monitored to balance the kinetic and potential energy, and the resonant frequency is automatically adjusted.^18^ This configuration is depicted in a schematic representation of these processes in Figure 1.^18^

Previous applications of RAM have been primarily focused on powder mixing, for example, in pharmaceuticals or dye utilization.^18,19,21,22^ However, in addition, RAM technology has been used for the solvent-free synthesis of metal–organic frameworks, large-scale production of lipidic materials, and the chemical coating of carbon fiber powders.^18,19,21,22^ These latter examples show the potential advancements that could be made by using this alternative mixing technique in polymer processing.

To the best of our knowledge, this is the first reported case of the use of RAM to produce polymers with minimal or no use of solvents or organometallic reagents, achieving a significantly reduced reaction time, thus developing a green and robust synthesis route that also uses naturally occurring monomers and renewable terpene alcohol initiators. An additional target was to exploit RAM as a method of synthesis scale-up and to investigate a small library of terpene-polylactides for protein encapsulation and release. The product material’s performance related to this property was then measured using a modified, high-throughput BCA assay, and the polymer characterization for the materials used in this assay is shown in S1–S3.

Although the RAM used in this work was a laboratory-scale mixer (LabRAM II) capable of mixing at volumes of 1 kg, there are commercially available higher volume capacity systems that operate up to 420 kg for large batch production scale and custom-built continuous acoustic mixers.^24^ This demonstrates the industrial scale at which the chemistry reported here could potentially be conducted.

## Materials and Methods

### Materials

Farnesol (95%), L-carveol (mixture of cis and trans isomers, ≥95%), geraniol (98%), trans-*p*-menth-6-ene-2,8-diol (99%), benzoic acid (≥99.5%), and 3,6-dimethyl-1,4-dioxane-2,5-dione (LA) (99%) were purchased from Sigma-Aldrich (AU). The commercial poly(lactic acid) polymer was purchased from Akina Polymers (catalog number AP287). Diazabicyclo[5.4.0]undec-7-ene (DBU) (99%) was purchased from Alfa Aesar. Catalysts were dried with activated 3 Å molecular sieves prior to use. All other materials were used as received. Acetonitrile (99.9%, HPLC gradient grade) and KCl (AR grade) were purchased from Fisher. *trans*-2-[3-(4-*tert*-Butylphenyl)-2-methyl-2-propenylidene] malononitrile (DCTB, ≥99%) was purchased from Sigma-Aldrich. Ultrapure water was obtained from a Milli-Q system. Dry dichloromethane was purchased from Sigma-Aldrich (AU) and stored under activated 3 Å molecular sieves. Standard solvents (diethyl ether, petroleum ether, etc.) were used without further purification.

### General Synthetic Procedures

#### Ring-Opening Polymerization of Lactide with Magnetic Stirrer Mixing

The initiator and monomer (lactide) (1 g) were weighed into a predried vial equipped with a dry stirrer bar. The monomer and initiator ratios were varied depending on the target polymer. Dry DCM (varied from 5 to 0 mL) was added, and the vial was capped. The mixture was allowed to fully solubilize with magnetic stirring. A sample was taken for ^1^H NMR analysis (*t* = 0). DBU (3 mol % wrt. [monomer]) was added as an organic catalyst. The reaction was stirred magnetically at room temperature (25 °C) for 30 min. The reaction was quenched by the addition of benzoic acid (5 mg/mL) and purified by precipitation into a cold 50:50 mixture of diethyl ether: 40–60 °C petroleum ether (40 mL). The resulting mixture was centrifuged at 4000 rpm for 10 min before decanting the organic solvent. The residue was dried in a vacuum oven to obtain the final oligomer as either a white solid or a viscous orange liquid.

#### Ring-Opening Polymerization of Lactide with Sonication Bath

The initiator and monomer (lactide) (1 g) were weighed into a predried vial. The monomer and initiator ratios were varied depending on the target polymer. Dry DCM (varied from 5 to 0 mL) was added, and the vial was capped. The mixture was allowed to fully solubilize with a vortex mixer. A sample was taken for ^1^H NMR analysis (*t*= 0). DBU (3 mol % wrt. [monomer]) was added as an organic catalyst. The reaction vial was placed inside the water bath of a Grant Ultrasonic Bath XUBA1 and allowed to sonicate at room temperature (25 °C) for 30 min. The reaction was quenched by the addition of benzoic acid (5 mg/mL) and purified by precipitation into a cold 50:50 mixture of diethyl ether: 40–60 °C petroleum ether (40 mL). The resulting mixture was centrifuged at 4000 rpm for 10 min before decanting the organic solvent. The residue was dried in a vacuum oven to obtain the final oligomer as either a white solid or viscous orange liquid.

#### Ring-Opening Polymerization of Lactide with a Sonicator Probe

The initiator and monomer (lactide) (1 g) were weighed into a predried vial. The monomer and initiator ratios were varied depending on the target polymer. Dry DCM (varied from 5 to 0 mL) was added, and the vial was capped. The mixture was allowed to fully solubilize with a vortex mixer. A sample was taken for ^1^H NMR analysis (*t*= 0). DBU (3 mol % wrt. [monomer]) was added as an organic catalyst. The reaction vial was placed inside the water bath, and the thermocouple and sonifier tip were immersed in the water bath. Branson Digital Sonifier 250 was set at 50% amplitude with a maximum operating temperature of 40 °C for 30 min. The reaction was quenched by the addition of benzoic acid (5 mg/mL) and purified by precipitation into a cold 50:50 mixture of diethyl ether: 40–60 °C petroleum ether (40 mL). The resulting mixture was centrifuged at 4000 rpm for 10 min before decanting the organic solvent. The residue was dried in a vacuum oven to obtain the final oligomer as either a white solid or viscous orange liquid.

#### Ring-Opening Polymerization of Lactide Utilizing Resodyn

The initiator and monomer (lactide) (1 g) were weighed into a predried plastic vial with a suitable screw cap. The monomer and initiator ratios were varied depending on the target polymer. Dry DCM (varied from 5 to 0 mL) was added, and the vial was capped. The mixture was allowed to fully solubilize with a vortex mixer. A sample was taken for ^1^H NMR analysis (*t*= 0). DBU (3 mol % wrt. [monomer]) was added as an organic catalyst. The reaction vials were immediately placed in a LabRam II Resodyn acoustic mixer. The samples were mixed at an acceleration of 90 g for 30 min. The reaction was quenched by the addition of benzoic acid (5 mg/mL) and purified by precipitation into a cold 50:50 mixture of diethyl ether: 40–60 °C petroleum ether (40 mL). The resulting mixture was centrifuged at 4000 rpm for 10 min before decanting the organic solvent. The residue was dried in a vacuum oven to obtain the final oligomer as either a white solid or viscous orange liquid.

### Analytical Techniques

#### Nuclear Magnetic Resonance (NMR)

^1^H and ^13^C NMR spectra were recorded on a Bruker DPX 400 NMR spectrometer operating at 400 MHz (^1^H) and 100 MHz (^13^C). Samples were prepared in CDCl_3_ (stated otherwise if applicable ), and chemical shifts, assigned in parts per million (ppm), are referenced to this solvent peak. All spectra were obtained at ambient temperature (22 °C ± 1). See detailed resonance assignment below. Details of NMR analysis are in Supporting Information.

#### Gel Permeation Chromatography (GPC)

GPC analysis was performed using an Agilent 1260 Infinity instrument equipped with a double detector with a light scattering configuration. A combination of PLgel 5 μm Mixed D and PLgel 3 μm Mixed E columns was used depending on the molecular weight of the oligomeric material. The system was operated at 35 °C, using THF as the mobile phase with a flow rate of 1 mL/min. GPC samples were prepared in HPLC-grade THF and filtered prior to injection. Analysis was carried out using Astra software. The number-average molar mass (*M*_n_) and dispersity (*Đ*) were calculated by using PMMA for the calibration curve.

#### Matrix-Assisted Laser Desorption/ionization Time-of-Flight Mass Spectrometry (MALDI-ToF)

MALDI-ToF was performed on a Bruker Autoflex instrument operated in positive ion reflectron mode. Each spectrum was accumulated from 5000 scans. Calibration was achieved using two different polyethylene glycol standards, with number-average molecular weights of 1500 and 3500 g mol^–1^, and was accurate to within 1 Da. Polymer samples and the matrix solution were prepared at a concentration of 10 mg mL^–1^ in acetonitrile. Sample and matrix solutions were mixed at a ratio of 1:2, and 0.5 μL of the mixed solution was spotted onto a polished steel MALDI target plate and allowed to dry for 5 min under standard conditions. 0.5 μL of a 0.05 M KCl solution was subsequently placed over the sample spot to act as the cation source and allowed to dry for 5 min under standard conditions. Sample analysis was accurate to within 2 Da and typically within <1 Da.

#### Fiber Loading Procedure

Azoalbumin (80 mg) was dissolved in 2.4 mL of deionized water. The solution was added to 9.6 mL of acetone and mixed at room temperature. Twenty-five mg of polymer was dissolved in 1.7 mL of acetone, and 0.3 mL of the protein stock solution was added. The polymer–protein solution was then added dropwise to a 100 mL beaker containing 20 mL of ethanol and 0.2 mL of 0.9% saline under stirring. After the complete addition of the polymer–protein solution, the resulting mixture was transferred to a 50 mL centrifuge tube and allowed to settle for 20 min. The reactant mixture then centrifuged at 3000 rpm for 1 min, and the liquid was drawn from the top and removed.

#### BCA Loading Assay Procedure

The reagent solutions used were as follows:

- Solution A: 5 g of dipotassium hydrogen phosphate (K_2_HPO_4_) and 0.5 g of bicinchoninic acid disodium salt (BCA-Na_2_) dissolved in 250 mL of deionized water.
- Solution B: 0.4 g of copper(II) sulfate pentahydrate (CuSO_4_·5H_2_O) dissolved in 250 mL of deionized water.
- Plain buffer: 5 g of K_2_HPO_4_ dissolved in 250 mL of deionized water.

Azoalbumin (25 mg) was dissolved in 25 mL of the plain buffer solution (concentration = 1 mg/mL). Serial dilution was performed to produce a set of standards with concentrations of 0.5, 0.25, 0.125, 0.0625, and 0.03125 mg/mL. 0.5 mL of each standard was taken and transferred to individual vials. 3.75 mL of solution A and 1.25 mL of solution B were added to each vial. Polymer fibers (75 mg) loaded with 3 mg of azoalbumin (total) were suspended in 7.5 mL of solution B in a 50 mL centrifuge tube. The suspension was then subjected to 75 g for 2 min in an acoustic mixer. 22.5 mL of solution A and 3 mL of plain buffer solution were then added. The mixture was maintained at a constant temperature and agitated at 400 rpm via orbital shaking. After the time point had passed, the agitation was stopped and 1 mL of the solution was taken; the aliquot was then filtered, and the solution was collected for analysis via absorbance at 562 nm (a correction factor to the concentration measured of 33/32 was applied after each time point). Meanwhile, 1 mL of plain buffer solution was added to the centrifuge tube, and agitation was resumed. This procedure was repeated in parallel for the polymer fibers that were prepared without azoalbumin. The amount of protein can be determined by the difference in absorbance between the sample and the control. This can then be plotted against time to show the decrease in the amount of protein present, which fits a linear model, and the exponential decay equation can be calculated.

## Results and Discussion

**Figure 2:**
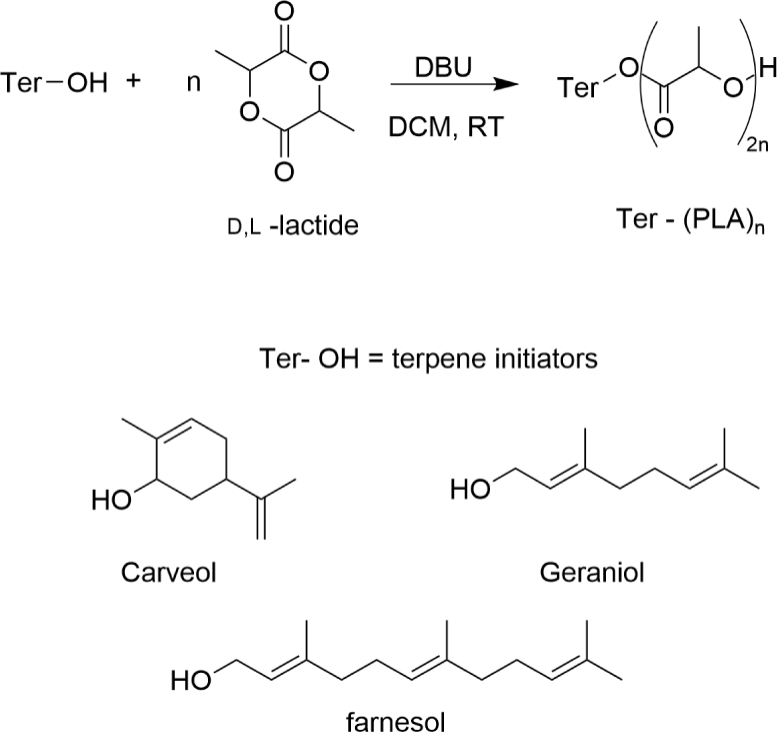
Reaction scheme for lactide ROP initiated by terpene alcohols and the molecular structures of the monoterpene alcohols used in the scale-up synthesis.

A proposed reaction scheme for the ROP of lactide monomer with functionalized terpene-alcohol initiators is shown in Figure 2, along with the molecular structures of example terpene alcohol initiators used in this scale-up study, having previously been reported to successfully initiate ROP.^5,15^

Initial studies were conducted on a 1 g monomer scale, and the monomer: initiator ([M]:[I]) was fixed at 5:1 ratio. The lactide monomer was solid at room temperature, while the terpene initiators were all liquids. As this study targeted the production of short polymer chain lengths, high concentrations of the initiator were required, which meant that the larger component of the reaction mixture was liquid. In these initial experiments, the initiator and the monomer are solubilized in varied amounts of DCM and allowed to fully dissolve. This solvent was added to ensure that the polymer product also solubilized during processing. 1,8-Diazabicyclo[5.4.0]undec-7-ene (DBU) was chosen as the reaction catalyst to ensure that the key headgroup and end-group functionalities were retained due to its mild activity/minimal side reactions.^5,23,24^

### Mixing via Magnetic Stirring

Initially, conventional magnetic stirring was adopted as the mixing technique to synthesize PLA using carveol as an initiator (see Figure 2 for molecular structure), as described in the Materials and Methods section. The amount of DCM solvent was varied to define the minimum amount of solvent needed for the process to be viable. The target DP was 5 at 100% conversion. Thus, to gauge initiator efficiency, this was compared to the conversion-corrected DP experimental results; i.e., the experimental GPC *M*_n_ was converted to the theoretical weight that would have achieved 100% conversion. See Table S1 for an example conversion-corrected DP_n_ and initiator efficiency calculation and Table 1 for the experimental data.

**Table 1:** Characterisation Data for PLA Polymers Synthesized Using Carveol as the Initiator, Magnetic Stirring, and Varying Amounts of DCM as Solvent for 30 Min^a^^b^

No	DCM (mL)	DP_n_^c^^,^^d^	Conv^e^ (%)	Conv^c^^,^^f^ Corr DP_n_	*M*_n_^g^ (g mol^–1^)	*M*_n_^d^ (g mol^–1^)	*Đ*^d^	Init^h^ Eff (%)
1	5	25	97	26	1600	3800	1.4	19
2	0.50	20	13	159	1700	3000	2.0	3
3	0.25	7	14	53	1300	1100	1.3	9
4	0.10	12	7	187	1200	1900	1.8	3
5	0	7	9	84	870	1100	1.2	6

a Reactions were on 1 g monomer scale, with [M]:[I] at 5:1 ratio, so targeting a DPn = 5.

b Mn is reported to be 2 s.f.

c DP values approximated to nearest whole values.

d Values generated by or calculated from GPC measurements.

e % conversion was calculated from ^1^H NMR by comparing integration of the LA monomer peak against the CH peak of the PLA chain.

f Predicted DP if polymerization had achieved 100% conversion from GPC data.

g *M*_n_ calculated from ^1^H NMR by comparing integrations of terpene alkene peaks to the CH peak of the PLA chain.

h Calculated by ratioing targeted and conversion corrected DP_n_ values.

The first entry shows the characterization data obtained when 5 mL was used in a typical ROP at a 1 g monomer scale. High conversion (97%) was achieved, and the ^1^H NMR spectrum confirmed the retention of the terpene alkene group, which would be useful for postpolymerization functionalization. GPC analysis showed a narrow polymer dispersity (*Đ*) and the presence of a single molecular weight peak distribution, indicating that polymerization was well-controlled. However, a much higher DP was observed than that targeted, resulting in an initiator efficiency of ∼20%, which suggested difficulty with the carveol initiating the ROP. Meanwhile, when the amount of DCM was reduced to be in the 0.5–0 mL range, there was a significant drop in the reaction conversion. This indicated that there was insufficient solubilization and mixing of the reagents to effectively induce polymerization, demonstrated by the reduction in initiator efficiency.

### Mixing via Sonication

Subsequently, mixing via sonication was explored as an alternative technique. Both an ultrasonic water bath and a sonication probe immersed in a water bath were utilized. Sonication uses sound waves to agitate the medium, which results in the growth and rapid collapse of microscopic bubbles.^25−27^ This rapid motion can result in efficient mixing, the generation of heat, and the formation of liquid jets.^26^ There are several examples of high-intensity ultrasound being used in polymerizations, including the radical polymerization of vinyl monomers and the ROP of cyclic siloxanes.^25−28^ This technique has also been applied for the ROP of cyclic lactones, such as ε-caprolactone or δ-valerolactone, and was shown to accelerate these polymerizations.^27^ From the previous literature studies, it was therefore hypothesized that sonication could be an appropriate mixing technique for the ROP of lactides. Thus, a series of reactions were conducted using this mixing mode, which were then quenched and purified using the methods described in the Materials and Methods section, and the resultant polymers were characterized by NMR and GPC. Initiator efficiency was gauged as shown in Table S1, and the experimental data are in Table 2.

**Table 2:** Characterisation Data for PLA Polymers Synthesized Using Carveol Initiator, the Sonicator Probe or Bath, and Varying Amounts of DCM as a Solvent for 30 Min^a^^b^

No	Sonic Type	DCM (mL)	DP_n_^c^^,^^d^	Conv^e^ (%)	Conv^c^^,^^f^ Corr DP_n_	*M*_n_^g^ (g mol^–1^)	*M*_n_^d^ (g mol^–1^)	*Đ*^d^	Init^h^ Eff (%)
_1_	Probe	0.50	18	90	18	2500	2700	1.3	27
2	Probe	0.25	20	62	25	2300	3000	1.3	20
3	Probe	0.10	29	74	30	3300	4400	1.4	17
4	Probe	0		0	0	0	0	0	0
5	Bath	0.50	51	79	23	2700	7500	1.5	22
6	Bath	0.25	23	80	11	1400	3500	1.8	45
7	Bath	0.10	50	68	22	2300	7400	1.4	22
8	Bath	0	0	0	0	0	0	0	0

a Reactions were on a 1 g monomer scale, with [M]:[I] at a 5:1 ratio, so targeting DPn = 5.

b Mn reported to 2 s.f.

c DP values approximated to nearest whole values.

d Values generated by or calculated from GPC measurements.

e % conversion was calculated from ^1^H NMR by comparing integration of the LA monomer peak against the CH peak of the PLA chain.

f Predicted DP if 100% conversion achieved from GPC data.

g *M*_n_ calculated from ^1^H NMR by comparing integrations of terpene alkene peaks to the CH peak of the PLA chain.

h Calculated by ratioing target and conversion corrected DP_n._ values.

It was observed from Table 2 that both methods of sonication resulted in high to moderate conversions being achieved, which were typically higher than those with magnetic stirring, as shown in Table 1. Similar to the magnetic stirring results, generally, there was a decrease in monomer conversion as the amount of solvent was decreased, and narrow *Đ* values were observed. In all cases, a much higher degree of polymerization was observed than the targeted molecular weight, which again suggested difficulty with the carveol initiating the ROP. This is indicated by the initiator efficiency being typically in the 20% region, suggesting better efficiency than that with magnetic stirring but still far from the target level. When no solvent was added to the reaction vials in this mixing mode, no ROP was observed. This suggested that the reagents were not sufficiently mixed for the reaction to take place. Finally, there seemed to be only a very small difference between the types of sonicator used, with the probe giving slightly improved yields but very similar initiator efficiencies.

### Mixing via RAM

These mixing strategies were then compared to the use of acoustic agitation to induce mixing in the form of a resonant acoustic mixer (RAM). In this case, the reagents, solvent, and catalyst were weighed into a predried plastic screw-cap vial and immediately placed into a LabRAM II Resodyn acoustic mixer. The samples were mixed at an acceleration of 90 g for 30 min. The reactions were then quenched and purified using the same methods as described in the materials and methods section, and the resultant polymers were characterized with NMR and GPC, where initiator efficiency was assessed as shown in Table S1, see data in Table 3.

**Table 3:** Characterisation Data for PLA Polymers Synthesized Using Carveol Initiator, RAM, and Varying Amounts of DCM as a Solvent for 30 Min^a^^b^

No	DCM (mL)	DP_n_^c^^,^^d^	Conv^e^ (%)	Conv^c^^,^^f^ Corr DP_n_	*M*_n_^g^ (g mol^–1^)	*M*_n_^d^ (g mol^–1^)	*Đ*^d^	Init^h^ Eff GPC (%)	Init^h^ Eff NMR (%)
1	5	9	96	9	1400	1400	1.5	55	55
2	4	3	96	3	1400	600	1.9	153	55
3	3	3	97	3	1200	610	2.1	152	67
4	2	7	96	7	1000	1100	1.4	73	81
5	1	2	95	2	700	460	2.0	218	124
6	0.75	3	98	3	1000	620	1.7	151	83
7	0.50	7	99	7	1200	1100	1.6	75	68
8	0.25	2	98	2	580	500	1.8	202	165
9	0.10	4	86	5	1300	790	2.5	94	53
10	0	3	88	4	1200	640	2.3	126	60
11	0	3	42	8	580	540	3.4	64	59

a Reactions on 1 g monomer scale, with [M]:[I] at 5:1 ratio, so targeting DPn = 5, with the exception of entry 11, which represents a control reaction, i.e., no terpene initiator included.

b Mn reported to 2 s.f.

c DP values approximated to nearest whole values.

d Values generated by or calculated from GPC measurements.

e % conversion was calculated from ^1^H NMR by comparing integration of the LA monomer peak against the CH peak of the PLA chain.

f Predicted DP if polymerization had achieved 100% conversion from GPC data.

g *M*_n_ calculated from ^1^H NMR by comparing integrations of terpene alkene peaks to the CH peak of the PLA chain.

h Calculated by ratioing target- and conversion-corrected DP_n_ values.

Overall, the results in Table 3 showed that RAM is an attractive alternative mixing strategy that offers considerable potential processing benefits for ROP manufacture when compared against conventional magnetic stirring and the use of sonication. The data in Table 3 show that a high monomer-to-polymer conversion was observed for all reactions. A slight decrease in monomer conversion was observed when using 0.1 and 0 mL DCM (86% and 88%, respectively), but this was still significantly higher than achieved with the alternative stirring methods that were applied. This suggested that RAM could be a more efficient and sustainable alternative mixing strategy, as it allowed the toxic DCM solvent to be reduced or potentially eliminated.

Additionally, the isolated polymers were much closer to the theoretically predicted targeted molecular weight, suggesting that the mixing levels were much higher, which in turn allows carveol to function more efficiently as an initiator. This was evidenced by the fact that all of the initiator efficiencies were above 55%, where the optimum value would be 100%. However, some discrepancies were observed (i.e., values greater than 100%), and examples of broadening of the molecular weight distribution were noted, both of which suggested that this mixing mode also allowed any entrained water to initiate more efficiently.

In the GPC data, a number of cases have initiation levels greater than 100%, which was attributed to either/both the accuracy of the GPC measurement for such low molecular oligomers (i.e., they are measured against methyl methacrylate standards with a calibration range from 545 to 29120 g mol^–1^ and part of the distribution merged with the solvent peak) and the increased mixing also allowing water to initiate chains more easily. The first hypothesis was supported by the calculation of the initiator efficiency using the NMR-generated values (see Table 3), which measures molecular weight via ratioing specific integrals from the terpene end-group and main chain. By using these figures, we see that all but 2 of the entries in Table 3 have initiator efficiencies less than 100%. The latter conclusion was supported by the result from the control reaction, i.e., no terpene initiator included (Table 3, Entry 11), which had no terpene initiator present, and so the formulation within the vial contained only the lactide and DBU catalyst. Thus, it would be expected that no ROP would occur; however, this was not the case, which suggested that the ROP must be proceeding either by trace amounts of water in the system or by the DBU catalyst. The fact that this yield in this case was 42% again highlighted that the system was better mixed, such that all the reagents were more available to the monomer to achieve such a high yield. Thus, it is likely that the initiator efficiency is influenced by both of these factors when RAM is applied. This conclusion was supported by the work of Shakaroun et al., who described the ROP of β-lactones via initiation by DBU or TBD under neat conditions, thus demonstrating the dual basic and nucleophilic activity of DBU.^29^

Additionally, when compared to stirrer and sonication mixing, higher monomer-to-polymer conversions were observed with RAM processing, while producing polymers of a similar magnitude/dispersity, at room temperature and without an organometallic catalyst. This is significantly more favorable than the standard bulk ROP using Sn(Oct)_2_ as a catalyst at temperatures greater than 150 °C,^30^ thus allowing much lower environmentally impacting processing, as there is (a) no need for the use of environmentally polluting thermofluids, (b) no requirement for extended heating and cooling periods in the reaction, which are necessary to bring the reaction mixture to reaction temperature and back to safe handling conditions, and (c) no need for organometallic catalysts. This latter property essentially removes an entire process of catalyst manufacture, purification, and storage from the manufacturing cycle of these polymers.

The importance of these last two observations was further exacerbated by the speed of the chemistry. In this case, the RAM chemistry has essentially reached quantitative conversion in 30 min. This significantly outperformed the stirred, hot oil ROP-based reactions (Table 1) that utilize the typical “standard” polymerization laboratory methodology conducted by polymer researchers. However, the key aims of this work were to demonstrate that we have developed a more sustainable process for conducting ROP reactions. The main strategy adopted toward achieving this goal was to demonstrate that this process could be successfully conducted either: (a) in the absence of organometallic catalysts, but more preferably (b) catalyst-free. Therefore, no experiments with organometallic catalysts were included in this study. Nevertheless, comparison of the magnetic stirring data reported in this study to that in the literature showed that it was consistent with prior publications by the authors, which followed the laboratory-scale, conventionally heated (hot oil), Sn(Oct)_2_ catalyzed, bulk ROP processing caprolactone, a very similar reaction system, with the aid of the changes in dielectric properties. This prior study showed that quantitative conversions were only achieved after 4.5 to 5 h of stirred processing.^31,32^

### Scale-Up of Terpene-Initiated ROP of Lactide with RAM Technology

The robustness of this technique at a larger scale was then investigated by scalingup the polymerization reactions from 1 to 20 g of lactide monomer, in order to examine the robustness of this synthesis method. Scale-up of larger molecular weight polymers was also attempted to investigate the influence of the molecular weight on the initiator efficiency achieved.

To achieve this demonstration that other terpene alcohols could be utilized with RAM on a larger scale, a small library of terpene-initiated polyesters was synthesized. These ROPs targeted producing two different polymer chain lengths; i.e., the monomer: initiator ratio was fixed at either 5:1 or 30:1, using three different terpene headgroups. The chosen initiators, see Figure 2, had all previously been observed to successfully function as initiators for the ROP of lactide.^5^ They were selected to demonstrate that different molecular structures could be used with RAM, including a cyclic and shorter/longer length linear structure. Additionally, DBU (3 mol %) was used as the reaction catalyst. The reactions were conducted in the same manner as previously described but utilized a much larger 500 mL reaction vessel for the 20 g scale reactions. From the results for characterization data shown in Table 3, it was decided that a minimum amount of DCM was to be used in order to ensure diffusivity within the system; i.e., all the experiments down to 0.25 mL of DCM for a 1 g reaction gave a *Đ* < 2; therefore, this was defined as the minimum level of solvent required; thus, to keep v:v% of the percentage solvent the same, 5 mL of DCM was used for a 20 g scale reaction. The larger reaction vessel allowed sufficient headspace in the reactor during the reaction for the volatile DCM solvent. The characterization and initiator efficiency (see Table S1) data of the resulting polymers are shown in Table 4. The analyzed NMR data for these polymers are shown in the Analytical Data section, and example ^1^H NMR for these materials has been included in Figures S1–S6.

**Table 4:** Characterisation Data for PLA Polymers Synthesised Using Three Terpene Alcohol Initiators and RAM^a^^b^

Target polymer	DP_n_^c^^,^^d^,	Conv^e^ (%)	Conv^f^ CorrDP_n_	*M*_n_^g^ (g mol^–1^)	*M*_n_^d^ (g mol^–1^)	*Đ*^d^	Init^h^ EffGPC (%)
Ger-(LA)_30_	23	96	27	3500	4100	1.5	100
Ger-(LA)_5_	3	83	3	590	600	3.0	130
Farn-(LA)_30_	27	92	26	4100	3900	1.4	100
Farn-(LA)_5_	7	100	5	1200	910	1.2	95
Carv-(LA)_30_	54	99	53	3600	7800	1.2	55
Carv-(LA)_5_	6	100	5	1000	930	2.3	95

a Reactions on a 20 g monomer scale using 5 mL DCM as a solvent for 30 min with [M]:[I] 5:1 and 30:1 ratios targeting DPn = 5 and 30, respectively.

b Mn reported to 2 s.f.

c DP values approximated to nearest whole values.

d Values generated by or calculated from GPC measurement.

e % conversion was calculated from ^1^H NMR by comparing integration of the LA monomer peak against the CH peak of the PLA chain.

f Predicted DP if polymerization had achieved 100% conversion from GPC data.

g *M*_n_ calculated from ^1^H NMR by comparing integrations of terpene alkene peaks to the C*H* peak of the PLA chain calculated from ^1^H NMR.

h Calculated by ratioing target- and conversion-corrected DP_n_ values and values quoted to nearest 5%.

For the majority of the 20 g polymerizations, both very high monomer conversions and narrow molecular weight distributions were observed, see Table 4. However, geraniol-(LA)_5_ had a decrease in conversion (83%), and both geraniol-(LA)_5_ and carveol-(LA)_5_ had broader polymer dispersities in the region of 2.3–3.0. This result was unexpected as it was hypothesized that the reactions which contained the highest concentration of terpene should have resulted in the most homogeneous reaction mixture as the dispersity issues should be minimized. However, these terpenes were both more viscous than the farnesol reagent. Hence, it was proposed that this physical property of farnesol would result in an increase in diffusivity and a more homogeneous polymer with a lower polydispersity. These results also supported the conclusion that the oligomers are so small that the resolution of the GPC peaks is more difficult due to overlap with residual monomer and more sensitive to small changes in the resulting predicted *M*_n_, as the DP 30 polymers typically had an initiator efficiency close to 100%. However, further work investigating the effects of drying all of the reagents needs to be conducted to determine if these broader *Đ* results are the result of the presence of entrained water.

For the small library of materials produced in this work, the RAM has demonstrated itself to be a robust and easily scalable technology that can be utilized with large volumes of liquid. LabRAM II used here is capable of mixing at volumes of 1 kg and is limited only by the use of a suitable reaction vessel that can withstand the pressures and acceleration of the technology. This, therefore, provides a potentially industrially scalable, sustainable combined mixing and reaction technique for the synthesis of biodegradable polyesters.

Finally, to gauge the increase in chemical sustainability exhibited by RAM processing, the process mass intensity (PMI) (i.e., total mass of raw materials (except water)/total mass of final product) was calculated for the RAM process and compared to a literature example that had produced the correct similar size of oligomers (i.e., <10000 g mol^–1^) using mechanical mixing.^24^ It was decided to compare this to the literature because the magnetic stirring and sonication processes in this study did not produce the targeted oligomers but exhibited low initiator efficiencies. The PMI calculation to compare the literature value to the RAM data is described in detail in S1, and the example calculation is detailed in Table S2. This relates to the synthesis part only, as the postprocessing used the same materials in the same quantities. The PMI calculations gave values of 1.3 for the RAM process and 5.1 for the magnetic stirring methods. Thus, the first scenario exhibits much higher chemical sustainability. The key difference in this area is the volume of solvent that is needed to get the appropriate materials’ molecular weights.

To further characterize the terminal functionality of the polymers, samples were analyzed by MALDI-ToF MS. MALDI is a soft ionization technique that primarily generates singly charged ions and is highly useful for the analysis of large molecules and macromolecules in the mass range of ∼600–20000 g mol^–1^. As analysis without a cation source led to complex spectra with a mixture of Li, Na, and K adducts of varied intensity, KCl was utilized as a cation source to favor the formation of M^+^K adducts.

Typically, the spectra of the smaller molecular weight materials, e.g., farnesol-(LA)_5_ and geraniol-(LA)_5_, showed no significant peaks above 800 *m*/*z* and showed no evident repeat unit patterns due to the presence of ions from the matrix. As such, they were ruled too low Mwt to be analyzed by MALDI. Rather, the polymers analyzed were larger terpene-(LA)_30_ polymers. These polymers produced spectra that all showed patterns of peaks with the 72 unit spacing expected from the LA repeat unit. Closer analysis of these patterns of peaks indicated that a mixture of polymers with differing combinations of α- and ω-termini was present in all samples, as shown in Figure S7. Several peak distributions were seen, one of which corresponded to polymers of the form terpene-(LA)_*x*_-hydroxyl, proving that all three terpenes investigated were functional initiators for the ROP of lactide. In addition, terpene-(LA)_*x*_-benzoate peaks and carboxyl-(LA)_*x*_-hydroxyl peak distributions were noted. The benzoate-terminated species would have arisen from the benzoic acid quench at the end of the reaction, which would not have been 100% efficient, given the presence of hydroxyl-terminated species. Similarly, the presence of α-carboxyl species indicated that water was present in all reactions and initiated some fraction of the ROP in all cases. A fourth distribution, corresponding to carboxyl-(LA)_*x*_-benzoate, was detected solely in geraniol-(LA)_30_ (Figure S7e,f). Due to differences in ionization efficiency, no assumptions as to the actual abundance of each chain type could be inferred from the spectra. The more detailed elucidation of the α- and ω-functions and calculation of the maximum DP_n_ observed in each spectrum of the terpene-(LA)_30_ polymers are given in Table S3.

### High-Throughput Encapsulation and Release Rate Evaluation of Proteins by Terpene-LA Polymers

Biodegradable polymers find common use in therapeutics, particularly for the controlled release of drugs or bioactive substances.^33,34^ To investigate how the terpene headgroups impact the release rate, a protein (azoalbumin) was encapsulated using a solution precipitation technique.^35^ Subsequently, the release of the protein was assessed using a modified bicinchoninic acid (BCA) assay, where the half-life of protein release can be obtained by fitting an exponential function to the fraction of protein remaining, assuming that this follows a first-order rate law. The data from these studies are included in Figures S8 and S9.

**Figure 3:**
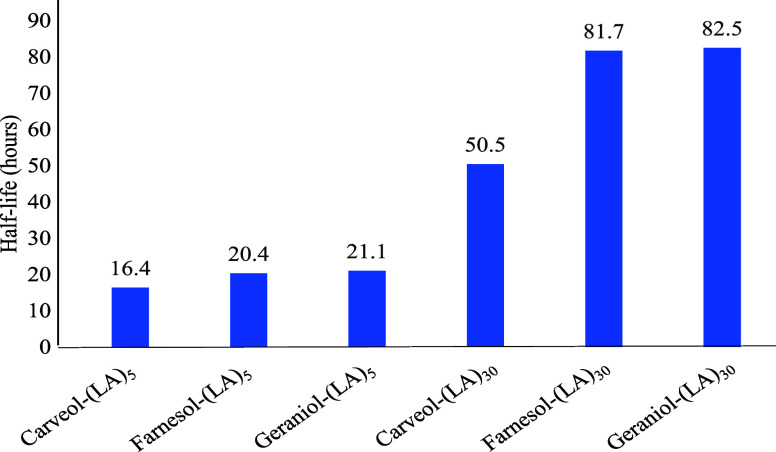
Half-lives (hours) of the terpene-initiated polymers loaded with azoalbumin. The exact half-lives (hours) are shown at the top of the bar chart. The assays accuracy in estimating the release half-life was assessed via experimental repeats conducted on a commercially purchased material (see Figure S10); these repeats demonstrated that the procedure standard deviation was 0.6, and the deviation from the mean was 0.4.

Furthermore, there were insufficient quantities of the polymers synthesized in this study to run multiple repeat protein release tests. The repeatability of the release protocol was determined by purchasing quantities of a commercially available polymer (Akina, catalog number AP287) and analyzing this using the same protocol. These data are shown in Figure S10 and showed high levels of repeatability between the repeats, giving confidence that the differences found in the terpene headgroup data were statistically significant. Figure 3 shows a bar chart comparing the half-lives for the terpene headgroup containing polymer.

Figure 3 shows that there is a significant increase in the half-life between the shorter chain lengths (*n* = 5) and the longer chain lengths (*n* = 30). This was expected because it was hypothesized that the lower molecular weight polymers would degrade faster due to higher levels of chain-end degradation. As the length of the polymer chain increases, the degradation of the polymer is slower and occurs through main chain degradation.

Interestingly, there was a similar trend in the half-life behavior exhibited by these terpene headgroup polymers at both molecular weights. The cyclic carveol-(LA)_5_ exhibited the shortest half-life, whereas the linear terpenes had longer half-lives that were similar to one another, likely reflecting the close resemblance of their molecular structures. At the shorter chain lengths (*n* = 5), minimal variations between the protein release half-lives were observed. Carveol-(LA)_5_ exhibited the shortest half-life of 16.4 h, whereas geraniol-(LA)_5_ had the longest half-life of 21.1 h. Meanwhile, the difference was much greater at longer chain lengths (*n* = 30), with the half-life of carveol-(LA)_30_ being 50.5 h and the half-life of geraniol-(LA)_30_ being 82.5 h. Thus, at this longer chain length, the influence of the headgroup was more significant. Thus, it was concluded that both the identity of the terpene headgroup and oligomer chain length had more effect on the protein release rate with longer chain lengths.

Although only a small sample of terpenes was investigated, the data in Figure 3 suggested that the linear structures of farnesol and geraniol can interact more strongly with the protein and delay the release. As opposed to the cyclic structure of carveol, a more rapid protein release was observed. This may suggest that the linear structures are more readily able to form microdomains or micelles that allow the protein to phase separate into this domain; thus, the release would be delayed as the whole polymer chain would have to degrade to achieve active release. The cyclic structure of carveol has reduced domain formation potential due to its more complex rigid structure that has a greater steric footprint localized at the chain end.

### Assessment of the Critical Aggregation Concentration

The influence of the headgroup structure was probed further by the assessment of the critical aggregation concentration (CAC) for these materials. The CAC is used to describe the concentration at which amphiphilic molecules within a specific system begin to self-assemble. At low concentrations, they exist as nonassociated species, i.e., existing as individual entities. However, as the concentration increases, the attractive/repulsive forces between the molecules cause self-aggregation to occur, resulting in the formation of monolayers and micelles.^36,37^

As these polymeric species can be regarded as polar oligomers with a hydrophobic-end group, making them act as amphiphiles/surfactants, the CMC of a polymer can be affected by several parameters. These include the overall molecule hydrophobicity, glass transition temperature (*T*_g_), extent of branching in the copolymer/headgroup, and ratio of the hydrophobic character of the oligomer: hydrophilic character of the headgroup.^38,39^ For example, it has been observed that with an increase in the length of the hydrophilic oligomer, the CMC tends to increase because this results in increased solubility of the polymer, so reducing the tendency to form aggregates.^40−42^ This is an important consideration for the final end-use application of an emulsion. For example, with respect to the stabilization/release of drugs in the body, as mimicked by the BSA study in this report, a lower CMC value is thought to indicate greater structural integrity of micelles upon severe dilution in a drug formulation.^43,44^ Hence, a copolymer with a lower CMC would be preferred owing to its *in vivo* stability and the small amount of copolymer that is required for micelle formation.

Thus, the CACs of carveol and geraniol headgroup terpene-PLA materials were measured and compared (a) with each other and (b) with a nonterpene headgroup (lauryl) to determine if they exhibited different aggregation characteristics based on the headgroup structure. These data are contained in Table 5.

**Table 5:** Comparison of CAC Values and Polymer Properties of PLA Synthesized Using Magnetic Stirring and Various Initiators^a^

Target polymer	CAC (μg mL^–1^)	*T*_g_ (°C)	*M*_n_^b^ Theo (g mol^–1^)	*M*_n_^c^ NMR (g mol^–1^)	*M*_n_^d^ GPC (g mol^–1^)	*Đ*^d^	Init^e^ Eff GPC (%)
Car-(LA)_54_	156	21.6	4500	7900	8400	1.6	55
Car-(LA)_34_	181	25.3	3000	5000	7900	1.4	40
Car-(LA)_23_	204	15.5	1600	3500	4700	1.5	35
Car-(LA)_15_	184	19.5	870	2300	3800	1.4	20
Ger-(LA)_34_	170	31.4	4500	5000	8200	1.3	55
Ger-(LA)_26_	154	31.4	3000	3900	5700	1.4	50
Ger-(LA)_24_	141	–9.3	1600	3600	4300	1.3	35
Ger-(LA)_16_	127	23.0	870	2500	2800	1.3	30
LaA-(LA)_31_	437	*-*	3100	4700	6600	1.4	45
LaA-(LA)_24_	389	*-*	1600	3600	6900	1.5	20
LaA-(LA)_5_	395	*-*	910	910	3000	1.3	25

a All reactions were reacted until they reached 100% conversion.

b Theoretical molecular weight from stoichiometric ratios.

c *M*_n_ calculated from ^1^H NMR by comparing integrations of terpene alkene peaks to the C*H* peak of the PLA chain calculated from ^1^H NMR.

d Values generated by GPC measurement.

e Calculated by ratioing target- and conversion-corrected DP_n_ values.

The data in Table 5 show that the measured CAC values and polymer molecular/physical characteristics of polymers were made via standard stirring. Magnetic stirring was utilized in this section of the study due to the lack of availability of the RAM system. The figures show that, while all the polymerization succeeded in reaching 100% conversion, the target molecular weight was often not achieved, exhibiting GPC initiation efficiencies of less than 50%. The typical trend in initiator efficacy achieved was shown to be the same with all the terpenes used, in that the lower the molecular weight targeted, the lower this value became. For the terpene-PLA materials, the CAC value varied from 127 to 203 μg mL^–1^. Furthermore, the geraniol-initiated polyesters, which contained a 12-carbon-long unsaturated, linear chain headgroup with side branches, exhibited an overall trend that as the PLA chain length of the polymer decreases, there was a decrease in the CAC value (Table 5, entries 5 to 8). A similar but less well-defined trend was also displayed by the “nonterpenoid” C12, lauryl linear, fully saturated headgroup (Table 5, entries 9 to 11) which is linear and fully saturated. Meanwhile, the carveol-initiated polyesters presented no discernible trend and thus exhibited different aggregation behavior from the linear headgroups.

For the functional headgroup oligomeric structures in this study, they are proposed to have a hydrophobic terpene as the headgroup and a hydroxyl as the tail group (see Figure 3). Thus, as the data in Table 5 show, they have defined CAC values suggesting that this PLA oligomer is exhibiting some “weak” hydrophilic character to counterbalance the nature of the headgroup. Thus, we see the expected trend demonstrated by both the terpenoid and the “nonterpenoid” linear headgroup polymers, that as the length of the PLA weak hydrophile decreases, the polymer is more dominated by the nature of the hydrophobic headgroup. Therefore, a lower concentration of polymer would be required to form micelles as the material would now be less water-soluble and more readily form micelles.

Meanwhile, for the carveol-initiated polyesters, no discernible trend was observed in the CAC results. This suggested that the constrained structure of the carveol headgroup may have restricted the formation of the micelles sterically. Furthermore, there are several other variables that could influence the CAC value.^45−48^ Thus, further research is needed to understand the additional parameters that are affecting the CAC/surfactancy behavior of such PLA functional headgroup oligomers with complex structured headgroups.

### Comparison of CAC Behavior of Terpene-Initiated Polymers with Nonterpenoid Polymers and Commercial Surfactants and Relation to Proteins Release Rate Data

While both C12 headgroup materials demonstrate the same trend in the CAC with PLA size change, the value of the geraniol-initiated polymers was found to be significantly lower than the lauryl-initiated equivalent. This was attributed to both the presence of branching and unsaturation in the headgroup and the potential change in the *T*_g_ of the materials (geraniol-(LA)_30_ = ∼ 31 °C). Thus, the geraniol materials would be predicted to be better amphiphiles for use in drug/personal care applications. Meanwhile, the carveol-LA materials presented CAC values that were similar to, but generally significantly higher than, those of the geraniol materials, but in this case, the *T*_g_ values appeared to stay more constant which perhaps resulted in the lack of a CAC trend. However, as carveol also contains branching, this may also contribute to the CAC value size. Thus, these higher CAC’s and the demonstrated lack of variance with the PLA oligomer length suggested a more inefficient packing of materials. This may explain why the BSA release was achieved more rapidly from the formulation, and the micelles are less stable.

Furthermore, comparing the PLA materials’ CACs to those of conventional surfactants, see a table of typical CAC for commercial samples in Table S4, it was shown that the terpene headgroup materials present similar CACs to rhamnolipid-like surfactants. However, these PLA materials can be synthesized and processed at a fraction of the cost, which is a major drawback for rhamnolipids. It would, therefore, be interesting to explore terpene polylactides as rhamnolipid replacements in surfactant applications.

## Conclusions

The work reported here has demonstrated the enormous potential of resonant acoustic mixing for solvent-free polymer synthesis. The ring-opening polymerization of lactide has been achieved with minimal to no solvent at room temperature, achieving high monomer conversion without the need for high reaction temperatures or metal catalysts, which are usually required for bulk polymerization of lactide. The potential scale-up amenability of the resonant acoustic mixer has also been demonstrated on a 20 g scale. The renewable polyesters produced here have been shown to effectively encapsulate a model protein, and the identity of the headgroup has a greater influence on the half-life as the polymer chain length increases. Other small molecules, such as dyes or enzymes, could be encapsulated to mimic these materials, acting as potential drug carriers for a range of biomedical applications.

## Supporting Information Available

It contains details of the peak assignments from NMR analysis conducted upon the polymers produced; tables containing examples of the calculations used for defining the degree of polymerization, the reaction initiator efficiency, the process mass intensity, and the polymer chain-end functions from the MALDI analysis. example proton NMR spectra for the polymer products synthesized; example MALDI-ToF mass spectra with peak assignments; plots of the fraction of protein remaining vs time (hours) for the terpene-(LA)5 samples showing the linear regression and exponential decay equations; a table of critical aggregation concentration values for relevant commercially available compounds (PDF)
