# Supplementary material for: Rapid, Highly Sustainable Ring-Opening Polymerization via Resonant Acoustic Mixing
Source: ACS Sustain Chem Eng. 2025 Jan 31;13(5):1916–26. doi: 10.1021/acssuschemeng.4c06330 (PMC11816011; doi:10.1021/acssuschemeng.4c06330)
Supplement: Supplementary file 1 — sc4c06330_si_001.pdf [file sc4c06330_si_001.pdf]

Supporting Information for

Rapid, Highly Sustainable Ring Opening Polymerisation via Resonant Acoustic Mixing

Harriet R. Fowler[a,+], Riley O'Shea[b,+], Joseph Sefton[c], Shaun C. Howard[b], Benjamin W. Muir[b], Robert A. Stockman[a], Vincenzo Taresco[a], and Derek J. Irvine[c,*]

[a] *School of Chemistry, University of Nottingham, University Park, Nottingham, NG7 2RD*

[b] *CSIRO Manufacturing, Clayton, Victoria 3168, Australia*

[c] *Centre for Additive Manufacturing, Department of Chemical and Environmental Engineering, Faculty of Engineering, University of Nottingham, University Park, Nottingham, NG7 2RD*

\+ *These authors contributed equally.*

*Corresponding author's email address: *derek.irvine@nottingham.ac.uk*

Number of pages: 14

Number of supporting analytical data presentations: 3

Number of supporting tables: 4

Number of supporting figures: 10

Analytical data for polymers used in protein release assay.

Analytical data S1: Data from synthesis targeting carveol-(LA)$_5$ and carveol-(LA)$_{30}$:

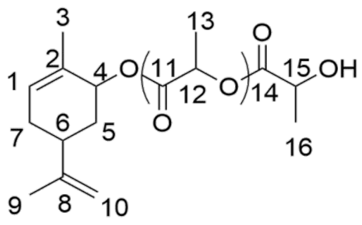

Carveol - (LA)$_5$

carveol-(LA)$_5$:

Car-(LA)$_6$ 100% conversion, calculated M_n from NMR: 1000 gmol^{-1}, M_n from GPC: 930 gmol^{-1} and Đ 2.3. **^{1}H NMR: (400 MHz, CDCl$_3$)** as a 1:1 mixture of diastereomers, δH = 5.77 (s, 1H, H1'), 5.63 (s, 1H, H1''), 5.56-5.47 (m, 1H, H4'), 5.30 -5.26 (m, 1H, H4''), 5.25 – 5.14 (m, 12H, H12), 4.79 – 4.71 (m, 4H, H10), 4.44 – 4.31 (m, 2H, H15), 2.06-1.94 (m, 4H, H5), 1.82 – 1.65 (m, 18H, H9, H3, H6, H7), 1.63 – 1.54 (m, 18H, H13), 1.52-1.49 (m, 6H, H16). **^{13}C NMR (100 MHz, CDCl$_3$)**

δC = 174.4 (C14), 169.6 (C11), 148.9 (C8), 133.7 (C2), 130.2 (C1), 128.5 (C10), 69.9 (C4), 69.0 (C12), 66.7 (C15), 30.6 (C9 or C3 or C6 or C7), 26.8 (C9 or C3 or C6 or C7), 23.7 (C9 or C3 or C6 or C7), 20.5 (C9 or C3 or C6 or C7), 20.2 (C16), 19.5 (C5), 16.52 (C13).

carveol-(LA)$_{30}$:

Car-(LA)$_{24}$ 99% conversion, calculated M_n from NMR: 3600 gmol^{-1}, M_n from GPC: 780 gmol^{-1} and Đ 1.2. **^{1}H NMR: (400 MHz, CDCl$_3$)** as a 1:1 mixture of diastereomers, δH = 5.77 (s, 1H, H1'), 5.63 (s, 1H, H1''), 5.56-5.47 (m, 1H, H4'), 5.30 -5.26 (m, 1H, H4''), 5.25 – 5.14 (m, 48H, H12), 4.79 – 4.71 (m, 4H, H10), 4.44 – 4.31 (m, 2H, H15), 2.06-1.94 (m, 4H, H5), 1.82 – 1.65 (m, 18H, H9, H3, H6, H7), 1.63 – 1.54 (m, 72H, H13), 1.52-1.49 (m, 6H, H16). **^{13}C NMR (100 MHz, CDCl$_3$)** δC = 174.4 (C14), 169.6 (C11), 148.9 (C8), 133.7 (C2), 130.2 (C1), 128.5 (C10), 69.9 (C4), 69.0 (C12), 66.7 (C15), 30.6 (C9 or C3 or C6 or C7), 26.8 (C9 or C3 or C6 or C7), 23.7 (C9 or C3 or C6 or C7), 20.5 (C9 or C3 or C6 or C7), 20.2 (C16), 19.5 (C5), 16.52 (C13).

Analytical data S2: Data from synthesis targeting geraniol-(LA)$_5$ and geraniol-(LA)$_{30}$:

geraniol-(LA)$_5$

Ger-(LA)$_3$ 83% conversion, calculated M_n from NMR: 590 gmol^{-1}, M_n from GPC: 600 gmol^{-1} and Đ 3.0. **^{1}H NMR: (400 MHz, CDCl$_3$)** δH = 5.36-5.30 (m, 1H, H9), 5.29-5.13 (m, 6H, H12), 5.10-5.06 (m, 1H, H4), 4.73 – 4.60 (m, 2H, H10), 4.44-4.33 (m, 1H, H15), 2.18-1.97 (m, 4H, H5, H6), 1.75-1.67 (m, 6H, H8, H1 or H3), 1.64-1.55 (m, 18H, H13), 1.54 – 1.49 (m, 6H, H16, H1 or H3). **^{13}C NMR (100 MHz, CDCl$_3$)** δC = 179.0 (C11), 169.6 (C14), 143.4 (C7), 133.8 (C2), 130.1 (C4), 128.5 (C9), 69.2 (C12), 66.7 (C15), 62.4 (C10), 29.0 (C8 or C1 or C3), 26.8 (C6 or C5), 25.2 (C8 or C1 or C3), 23.4 (C8 or C1 or C3), 20.3 (C16), 19.4 (C6 or C5), 16.8 (C13).

geraniol-(LA)$_{30}$:

Ger-(LA)$_{23}$ 96% conversion, calculated M_n from NMR: 3500 gmol^{-1}, M_n from GPC: 4100 gmol^{-1} and Đ 1.5. **^{1}H NMR: (400 MHz, CDCl$_3$)** δH = 5.36-5.30 (m, 1H, H9), 5.29-5.13 (m, 56H, H12), 5.10-5.06 (m, 1H, H4), 4.73 – 4.60 (m, 2H, H10), 4.44-4.33 (m, 1H, H15), 2.18-1.97 (m, 4H, H5, H6), 1.75-1.67 (m, 6H, H8, H1 or H3), 1.64-1.55 (m, 168H, H13), 1.54 – 1.49 (m, 6H, H16, H1 or H3). **^{13}C NMR (100 MHz, CDCl$_3$)** δC = 178.6 (C11), 169.7 (C14), 143.1 (C7), 131.9 (C2), 123.5 (C4), 117.5 (C9), 69.0 (C12), 66.7 (C15), 62.4 (C10), 29.0 (C8 or C1 or C3), 26.8 (C6 or C5), 25.7 (C8 or C1 or C3), 23.4 (C8 or C1 or C3), 20.1 (C16), 19.4 (C6 or C5), 16.4 (C13).

Analytical data S3: Data from synthesis targeting farnesol-(LA)$_5$ and farnesol-(LA)$_{30}$:

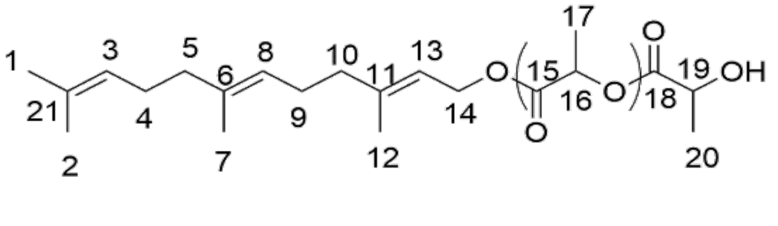

Farnesol- (LA)$_5$

farnesol-(LA)$_5$

Farnesol-(LA)$_7$ 100% conversion, M_n from ^{1}H NMR: 1200, M_n from GPC: 910 gmol^{-1} and Đ 1.2. **^{1}H NMR: (400 MHz, $CDCl_3$)** δH = 5.38-5.29 (m, 1H, H13), 5.29– 5.12 (m, 14H, H16), 5.13-5.08 (m, 2H, H8, H3), 4.72-4.61 (m, 2H, H14), 4.46 – 4.31 (m, 1H, H19), 2.07-2.00 (m, 8H, H4, H5, H9, H10), 1.74-1.68 (m, 6H, H1, H12), 1.65 – 1.54 (m, 42H, H17), 1.52-1.49 (m, 9H, H20, H2, H7). **^{13}C NMR (100 MHz, $CDCl_3$)** δC = 178.4 (C18), 169.6 (C15), 133.6 (C21), 130.3 (C6 and C11), 129.5 (C3), 128.5 (C13, C8), 69.0 (C16), 66.9 (C14, C19), 38.2 (C10 or C5), 32.7 (C10 or C5), 29.0 (C4 or C9), 26.8 (C4 or C9), 23.9 (C1),20.5 (C20), 19.6 (C2), 17.1 (C7, C12), 16.7 (C17).

farnesol-(LA)$_{30}$

Farnesol- (LA)$_{27}$ 92% conversion, M_n from ^{1}H NMR: 4100, M_n from GPC: 3900 gmol^{-1} and Đ 1.4. **^{1}H NMR: (400 MHz, $CDCl_3$)** δH = 5.38-5.29 (m, 1H, H13), 5.29– 5.12 (m, 58H, H16), 5.13-5.08 (m, 2H, H8, H3), 4.72-4.61 (m, 2H, H14), 4.46 – 4.31 (m, 1H, H19), 2.15-1.95 (m, 8H, H4, H5, H9, H10), 1.72-1.67 (m, 6H, H1, H12), 1.65 – 1.54 (m, 175H, H17), 1.52-1.49 (m, 9H, H20, H2, H7). **^{13}C NMR (100 MHz, $CDCl_3$)** δC = 178.4 (C18), 169.6 (C15), 143.3 (C11), 135.5 (C6), 131.3 (C21), 124.4 (C13 or C8), 123.5 (C13 or C8), 117.5 (C3), 69.0 (C16), 66.9 (C14, C19), 39.7 (C10 or C5), 39.5 (C10 or C5), 28.9 (C4 or C9), 26.6 (C4 or C9), 23.7 (C1),20.3 (C20), 19.4 (C2), 17.5 (C7, C12), 16.7 (C17).

*Table S1**.** Example Conversion Correction DP and Initiator Efficiency Calculation with Geraniol.* This example used the data from Table 1, Entry 3 which targeted a DP = 5 and achieved a GPC M_n of 1100 g mol^{-1} at 14% conversion. The molecular weight of Geraniol = 154 g mol^{-1} and that of repeat unit of lactide is 144 g mol^{-1}.

Title	M_n Values (g mol^{-1})	DP Values
DP Calculation Equation	GPC measured M_n	(GPC measured M_n - Head Group M_n)/ Repeat unit of lactide
Table 1 Entry 3 Worked Example	1100	(1100 – 154) / 144 = 7
Conversion Correction Equation	(Experimental M_n / Conversion achieved) x100	(Corrected M_n - Head Group M_n) / Repeat unit of lactide
Conversion Corrected Table 1 Entry 3 Worked Example	(1100 / 14) x 100 = 7857	(7857 – 154) / 144 = 53
Initiator Efficiency Equation	(Target DP/Conversion corrected DP) x 100	
Initiator Efficiency Table 1 Entry 3 Worked Example	(5/53) x 100 = 9	

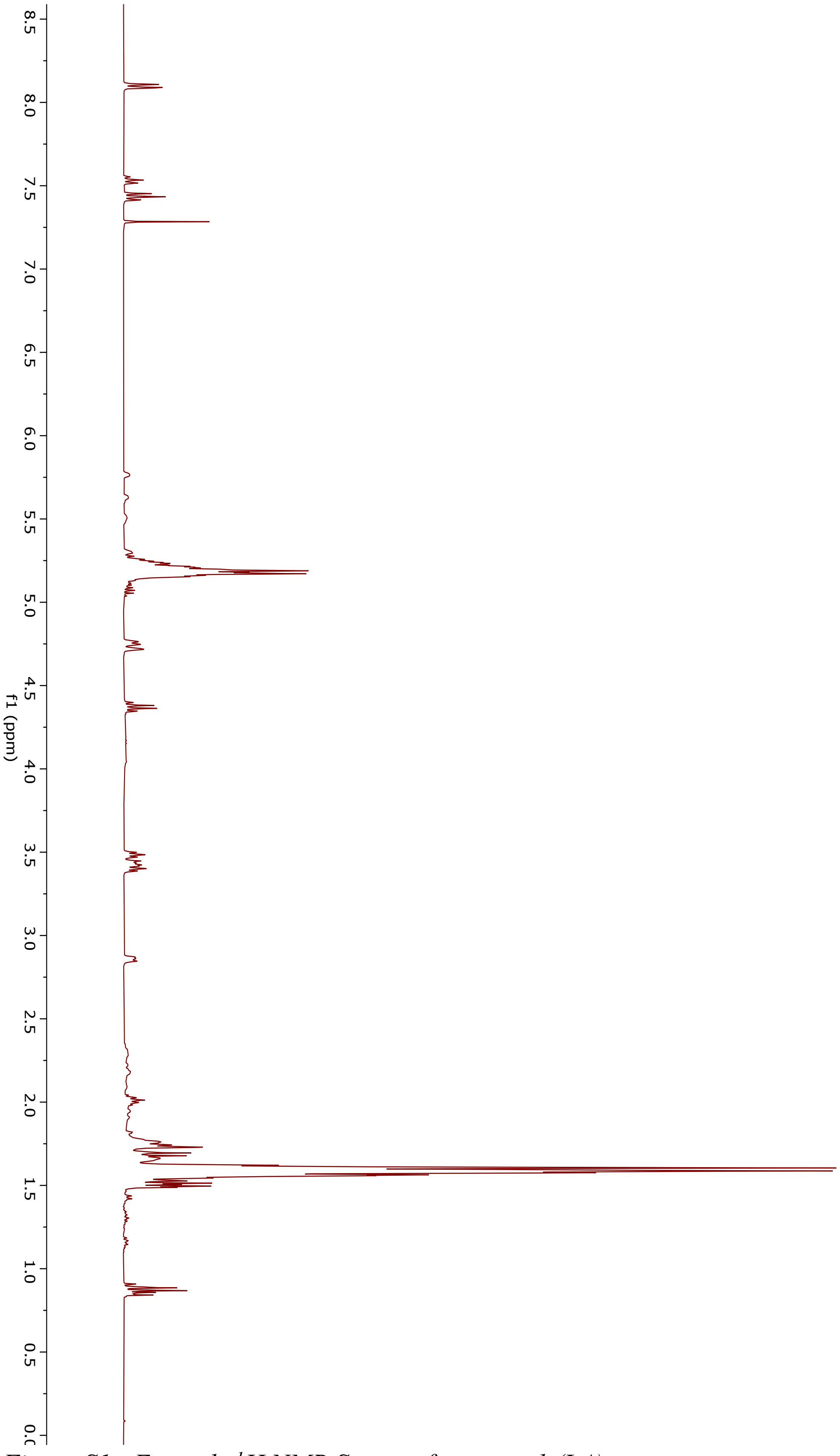

Figure S1: Example ^{1}*H NMR Spectra for carveol-(LA)*$_5$

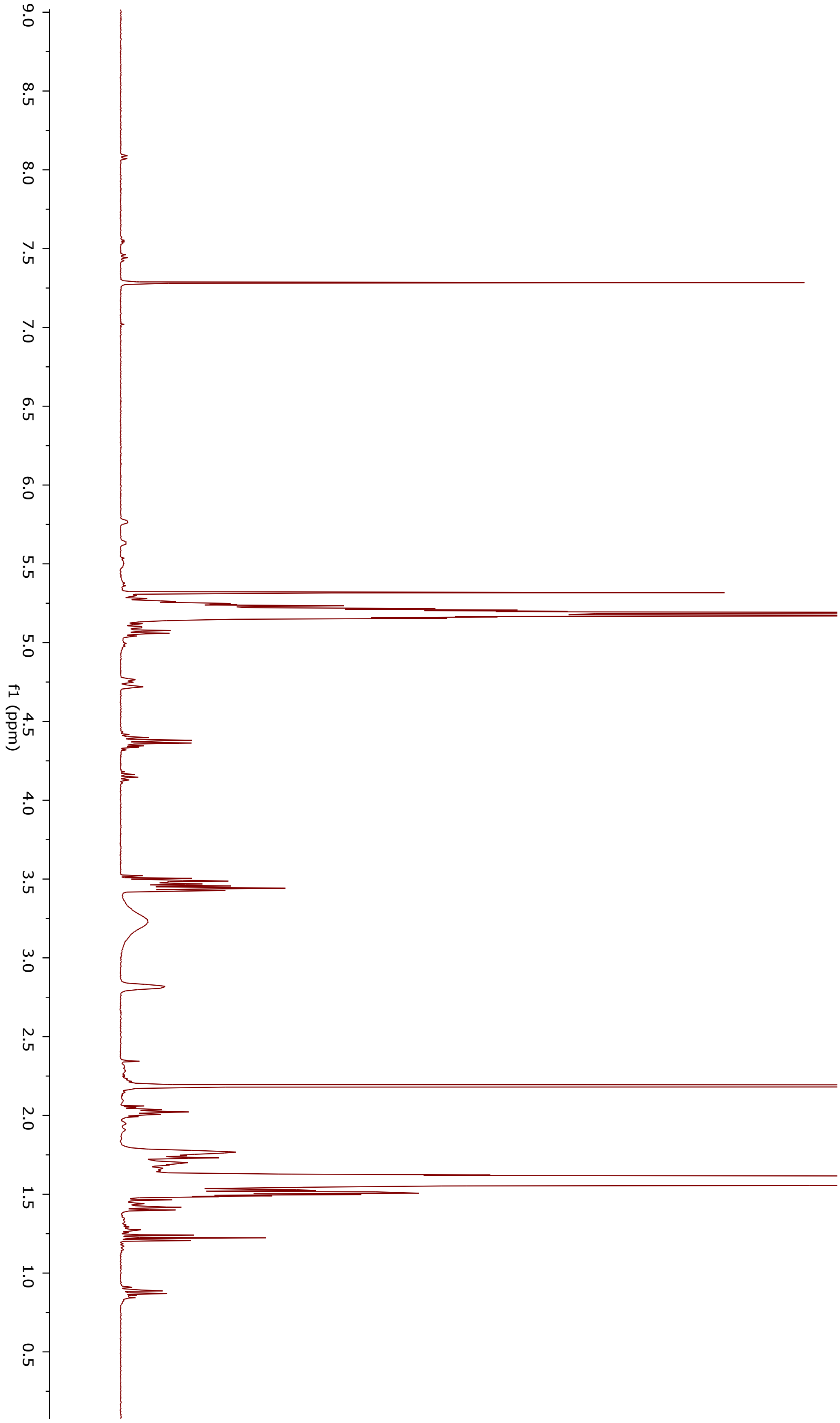

Figure S2: Example ^{1}H NMR Spectra for carveol-(LA)$_{30}$

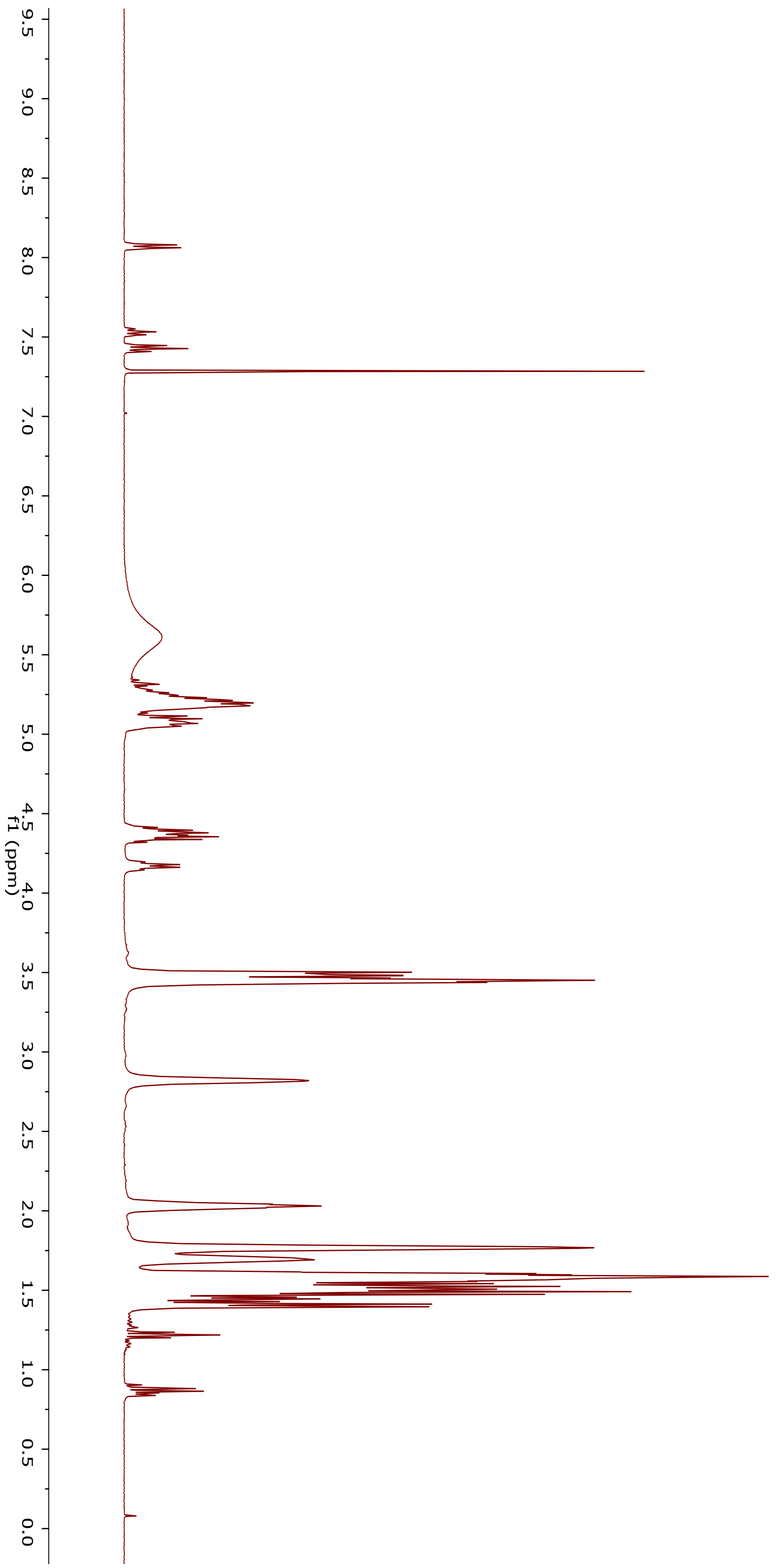

Figure S3: Example NMR Spectra for geraniol-(LA)$_5$

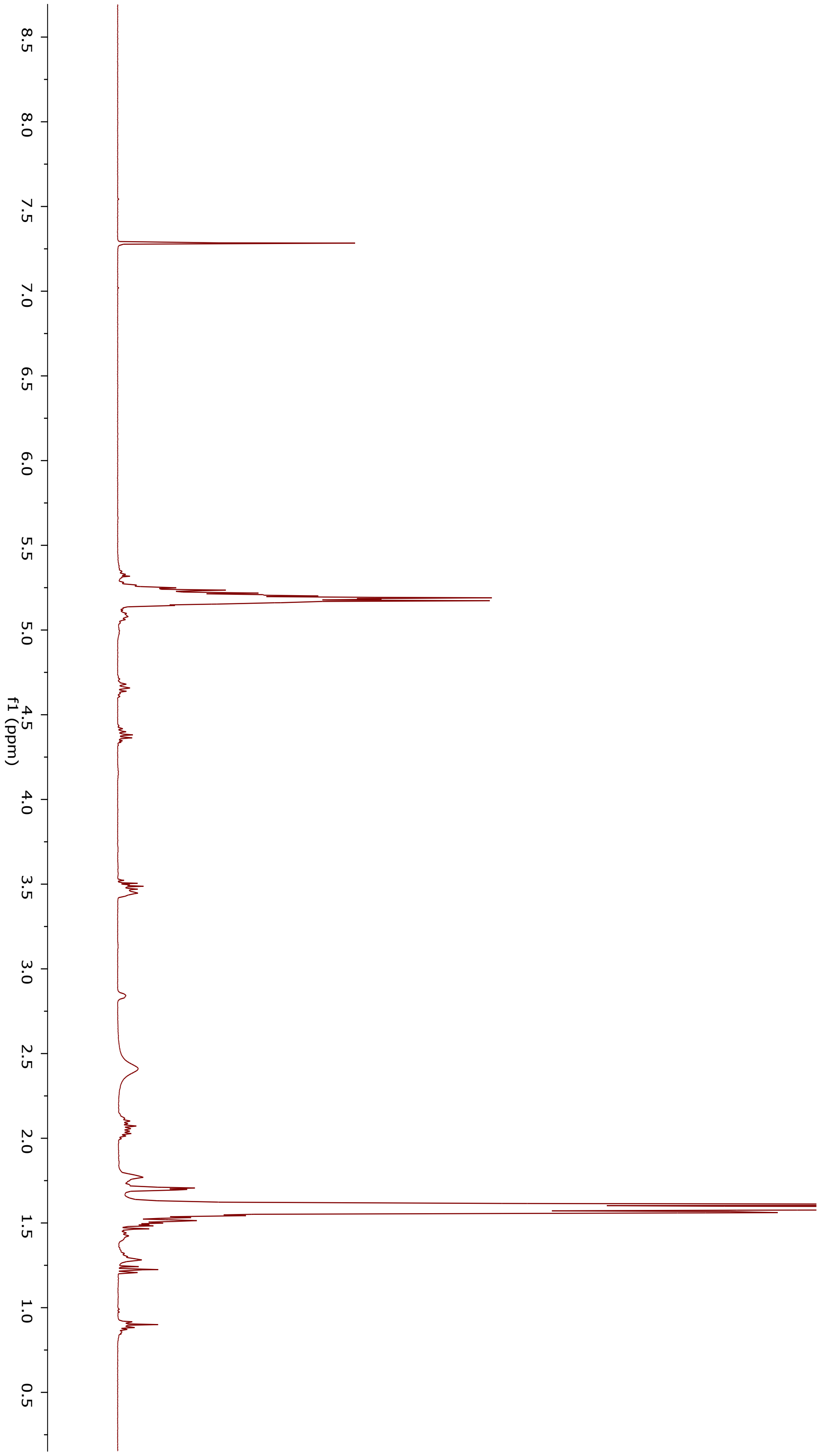

Figure S4: Example ^{1}H NMR Spectra for geraniol-$(LA)_{30}$

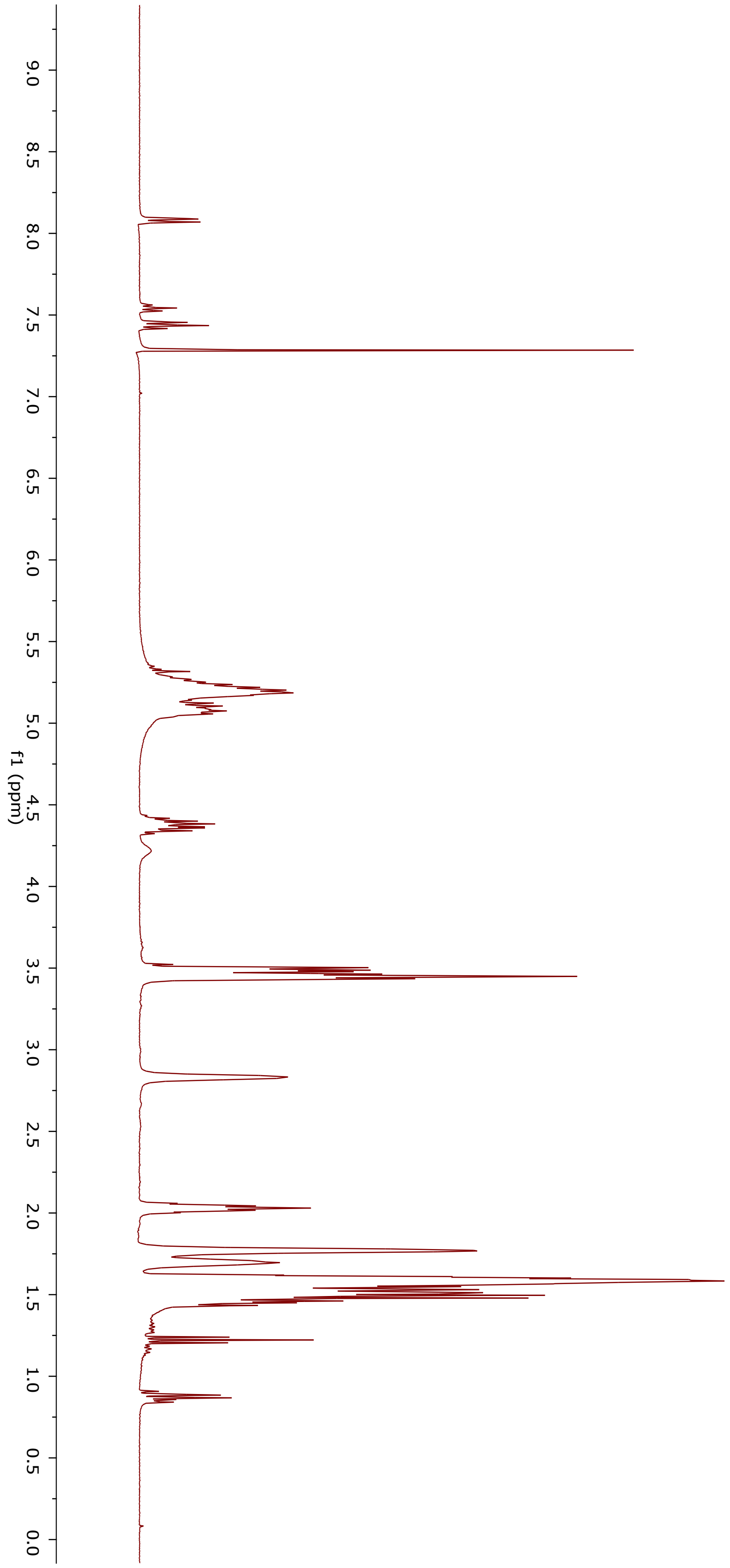

Figure S5: Example ^{1}H NMR Spectra for farnesol-$(LA)_5$

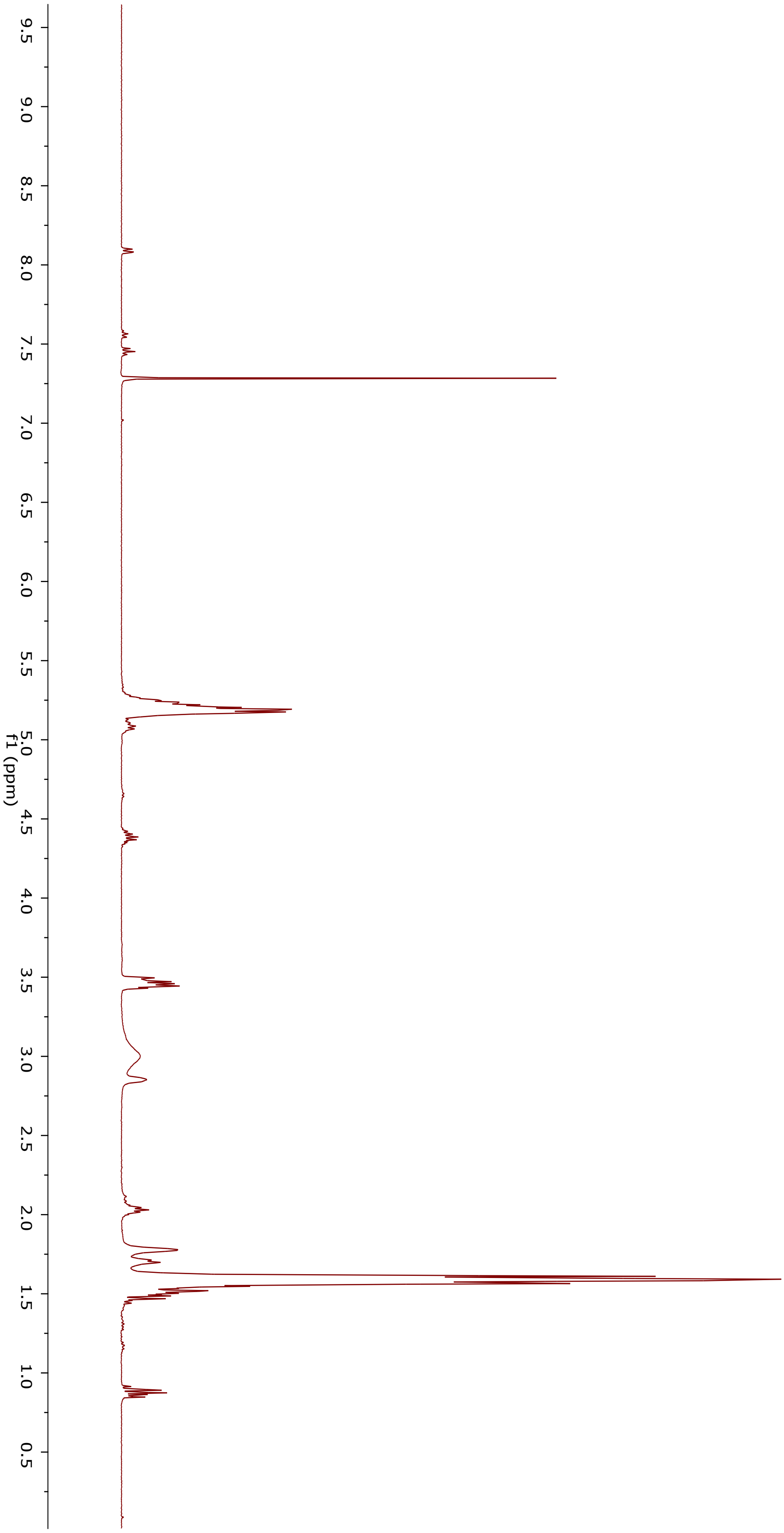

Figure S6: Example ^{1}H NMR Spectra for farnesol-(LA)$_{30}$

Calculations S1. Process Mass Intensity (PMI) calculations for a proposed 9 L reaction that reached the conversion of ~50% as recorded in Table 3.

Typically, the 5-L feed vessels were only filled with 4.5 L to prevent loss of containment when attaching to the apparatus. Thus, the calculation of the PMI was conducted using Equation 1 for the two scenarios detailed in the manuscript text: (a) where the monomer is a necessary part of the formulation as a viscosity modifier and (b) where it is simply a dilutant/solvent. These data are included in Table S1 below.

$$\text{PMI} = \frac{\text{Total mass of raw materials (except water)}}{\text{Total mass of final product}} \qquad \text{Equation 1}$$

Table S2. Masses of reagents and product and PMI calculation for a magnetic stirred and RAM polymerisation.[1]

**Magnetic Stirring Method Reagents**		**Weight in reaction vessel (g)**
PEG Initiator		38.9
Lactide		16.3
DCM		212.8
DBU		2.7
	**Reagent Total**	**268.0**
	**Product Total**	**52.5**
**RAM Method Reagents**		
Geraniol Initiator		4.25
Lactide		20.0
DCM		6.7
DBU		0.6
	**Reagent Total**	**31.5**
	**Product Total**	**24.3**
PMI Calculations		
Magnetic stirring method	268 / 52.5	5.1
RAM Method	31.5 / 24.3	1.3

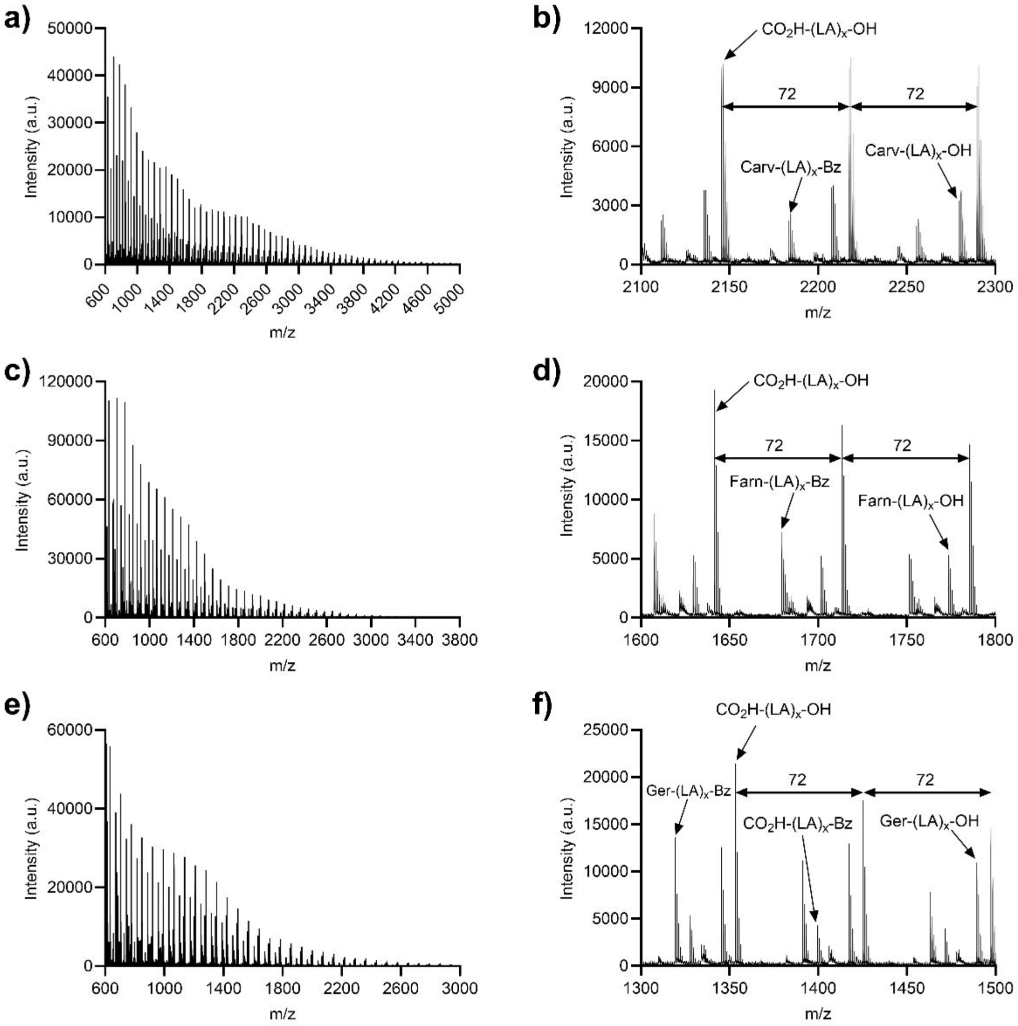

Figure S7: Example MALDI-ToF mass spectra and excerpts with peak assignments. Carv-$(LA)_{30}$ (a and b), Farn-$(LA)_{30}$ (c and d), and Ger-$(LA)_{30}$ (e and f). All labelled peaks are monopotassium adducts.

Table S3: Calculation of chain-end functions and DP_n for polymers from MALDI-ToF MS spectra. The chosen peaks are exemplar and not necessarily the highest m/z value for a given species. Monoisotopic masses utilised (in Da): K = 39.96, H = 1.01, O = 16.00, benzoate (Benz) = 121.03, LA = 72.02, carveol fragment (C) = 151.11, farnesol fragment (F) = 221.19, geraniol (G) fragment = 153.13. DP values have been quoted to nearest whole number.

Sample	M^+K peak (m/z)	Minus K (Da)	Head group	End group	DP_n LA	DP_n Lactide	Residual (Da)
C-(LA)$_{30}$	4513.50	4474.54	C	H	60[a]	30[a]	0.62
	1857.00	1818.04	OH	H	25	13	-0.73
	1823.51	1784.55	C	Benz	21	11	-0.31
F-(LA)$_{30}$	2793.82	2754.86	OH	H	38[a]	19[a]	-0.37
	2687.73	2648.77	F	Benz	32	16	1.59
	2709.92	2670.96	F	H	34	17	-0.26
G-(LA)$_{30}$	2937.96	2880.99	OH	H	40[a]	20[a]	-0.21
	1489.53	1450.57	G	H	18	9	-0.64
	1471.67	1432.71	OH	Benz	18	9	-1.87
	1463.39	1423.43	G	Benz	16	8	-0.64

[a] Maximum DP_n calculated from the mass spectrum.

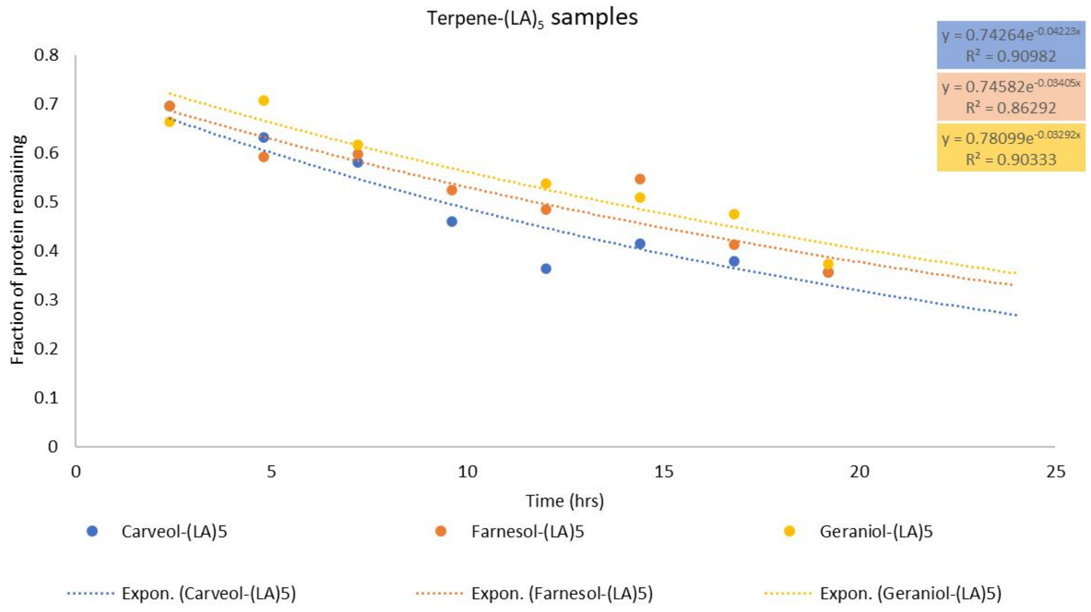

Figure S8: Plot of fraction of protein remaining vs. time (hours) for the terpene-(LA)$_5$ samples showing the linear regression and exponential decay equations.

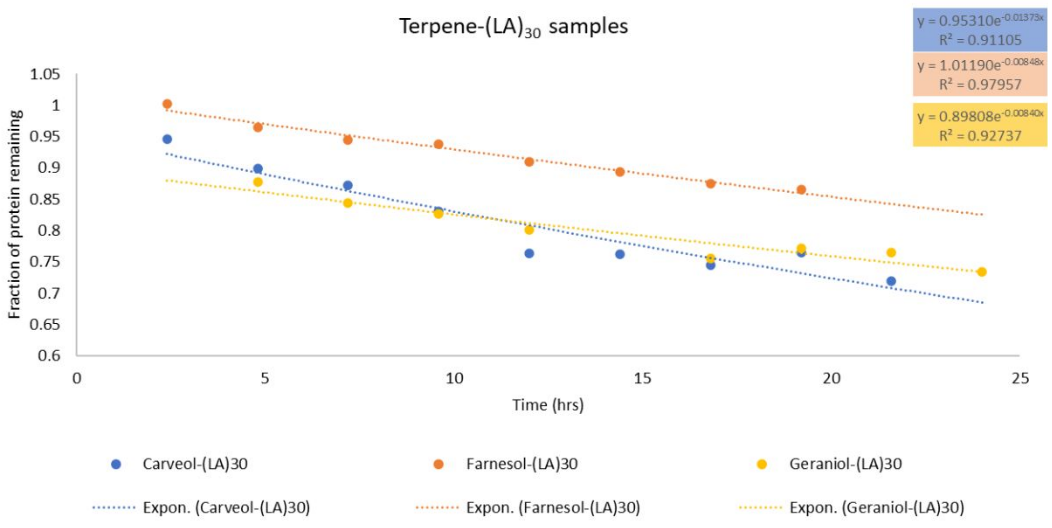

Figure S9: Plot of fraction of protein remaining vs. time (hours) for the terpene-(LA)$_{30}$ samples showing the linear regression and exponential decay equations.

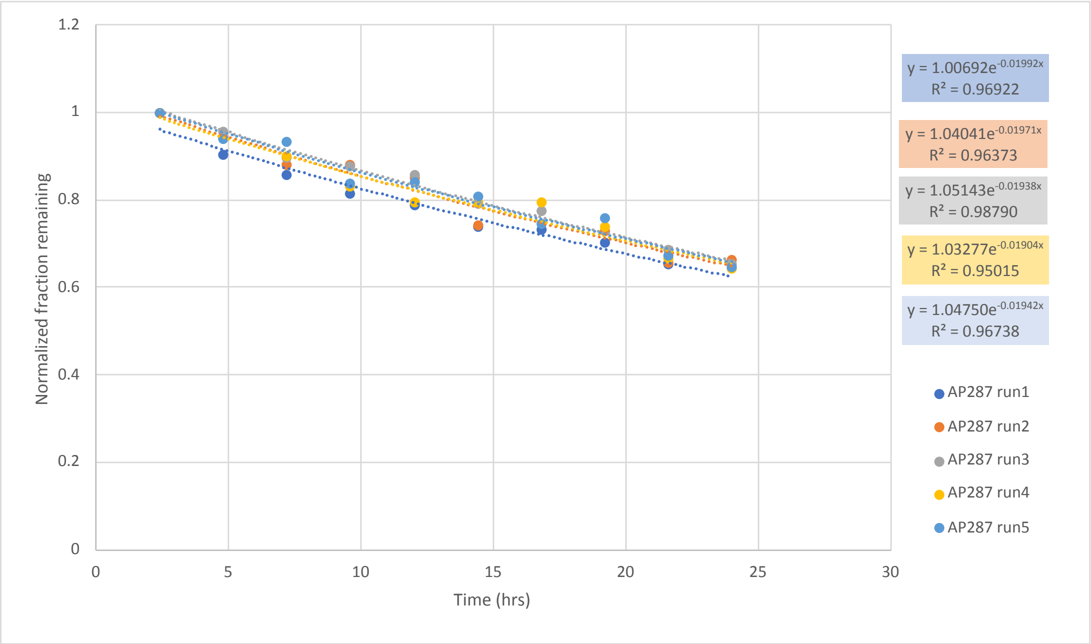

Figure S10: Plot of fraction of protein remaining vs. time (hours) for 5 independent experiments for a commercial obtained PLLA samples (Akina Polymers catalogue number AP287) showing the linear regression and exponential decay equations. The assays accuracy in estimating the release half-life was assessed using these repeats via calculating their standard deviation (0.6) and the deviation from the mean (0.4).

Table S4: CAC values of Example Commercial Surfactants

**Commercial surfactant**	**CMC ($\mu g\ mL^{-1}$)**
Sodium dodecyl sulphate	2451[2]
Sodium lauryl ether sulphate	222[2]
Cocamidopropyl betaine	30.8[2]

Sodium cocoyl alaninate	58.7[3]
Tween 80	12[4]
Brij 30	4.8[4]
Brij 56	2.4[4]
Rhamnolipid	275.4[5]

References

(1) Sherck, N. J.; Kim, H. C.; Won, Y.-Y. Elucidating a Unified Mechanistic Scheme for the DBU-Catalyzed Ring-Opening Polymerization of Lactide to Poly(Lactic Acid). *Macromolecules* **2016**, *49* (13), 4699–4713. https://doi.org/10.1021/acs.macromol.6b00621.

(2) El-Dossoki, F. I.; Gomaa, E. A.; Hamza, O. K. Solvation Thermodynamic Parameters for Sodium Dodecyl Sulfate (SDS) and Sodium Lauryl Ether Sulfate (SLES) Surfactants in Aqueous and Alcoholic-Aqueous Solvents. *SN Appl Sci* **2019**, *1* (933).

(3) Zhang, G.; Xu, B.; Han, F.; Zhou, Y.; Liu, H.; Li, Y.; Cui, L.; Tan, T.; Wang, N. Green Synthesis, Composition Analysis and Surface Active Properties of Sodium Cocoyl Glycinate. *Am J Analyt Chem* **2013**, *4* (09), 445–450. https://doi.org/10.4236/ajac.2013.49056.

(4) Cuzzucoli Crucitti, V.; Contreas, L.; Taresco, V.; Howard, S. C.; Dundas, A. A.; Limo, M. J.; Nisisako, T.; Williams, P. M.; Williams, P.; Alexander, M. R.; Wildman, R. D.; Muir, B. W.; Irvine, D. J. Generation and Characterization of a Library of Novel Biologically Active Functional Surfactants (Surfmers) Using Combined High-Throughput Methods. *ACS Appl Mater Interfaces* **2021**, *13* (36), 43290–43300. https://doi.org/10.1021/acsami.1c08662.

(5) Monnier, N.; Cordier, M.; Dahi, A.; Santoni, V.; Guénin, S.; Clément, C.; Sarazin, C.; Penaud, A.; Dorey, S.; Cordelier, S.; Rippa, S. Semipurified Rhamnolipid Mixes Protect Brassica Napus Against Leptosphaeria Maculans Early Infections. *Phytopathology* **2019**, *110* (4), 834–842. https://doi.org/10.1094/PHYTO-07-19-0275-R.
